# Agri-PIDNet-S: Lightweight semantic segmentation for field-structure and obstacle perception in seedling-stage cotton fields

**DOI:** 10.1371/journal.pone.0361992

**Published:** 2026-11-02

**Authors:** Yanlin Xin, Yongke Li, Wuchen Wei, Xinyu Cao, Bowen Mao, Yanhong Chen

**Affiliations:** 1 College of Computer and Information Engineering, Xinjiang Agricultural University, Urumqi, China; 2 Engineering Research Center of Intelligent Agriculture, Ministry of Education, Urumqi, China; 3 Xinjiang Agricultural Informatization Engineering Technology Research Center, Urumqi, China; Tsinghua University, CHINA

## Abstract

Reliable joint perception of crop rows, furrows, and obstacles is essential for autonomous inter-row operations in seedling-stage cotton fields. However, small and sparsely distributed plants, perspective-convergent rows, irregular furrow boundaries, and low obstacle pixel coverage jointly challenge fine-detail preservation, long-range structural modeling, and boundary discrimination under real-time constraints. This study formulated cotton-field perception as a four-class semantic segmentation task and constructed a dataset of 709 pixel-wise annotated images comprising background, crop rows, furrows, and obstacles. We developed Agri-PIDNet-S, a field-specific lightweight network based on PIDNet-S. A coordinate-aware multi-kernel inverted residual module (CA-MKIR) preserves shallow details and small-obstacle contours, a squeeze-and-excitation-enhanced deep-to-token module (SaE-D2T) models long-range row and furrow structures, and a boundary-guided window-attention refinement Bag (BWR-Bag) improves semantic–boundary fusion. The network was trained with a composite loss incorporating auxiliary boundary supervision. On the cotton-field test set, Agri-PIDNet-S achieved 92.37% mean intersection over union (mIoU) and 96.02% mean Dice coefficient (mDice) in the model-comparison experiment, while repeated training with three seeds yielded an mIoU of 92.35 ± 0.03%. Relative to PIDNet-S under the same comparison protocol, mIoU increased by 3.53 percentage points, while obstacle IoU improved from 79.60% to 88.67%. The model contained 8.048 million parameters, required 19.161 GFLOPs, and achieved 155.67 frames per second. Zero-shot external evaluation on tomato, watermelon, and maize fields yielded a structural mIoU of 86.29% for the background, crop-row, and furrow classes. Under the evaluated conditions, the task-oriented modifications achieved higher segmentation point estimates than PIDNet-S while maintaining computational efficiency; the external results provide preliminary evidence of cross-crop structural transfer.

## Introduction

Cotton is an important cash crop, and improving the mechanization and intelligent automation of cotton-field operations is essential for reducing labor requirements, increasing production efficiency, and supporting precision crop management. Agricultural robots and intelligent field machinery increasingly rely on machine vision to perform weeding, crop protection, field inspection, and inter-row operations [1–3]. For these operations, crop rows provide directional guidance, furrows indicate potentially traversable areas, and obstacles such as people, irrigation facilities, and field-monitoring equipment are directly related to operational safety. The seedling stage is particularly important because the cotton canopy has not yet fully closed and inter-row operations remain feasible. However, cotton plants at this stage are small and sparsely distributed, while missing plants, discontinuous rows, weeds, shadows, damaged plastic mulch, and complex soil textures introduce substantial visual interference. Representative cotton-field vision studies include plant detection, tracking, and counting [4], as well as visual navigation based on crop-row path perception [5]. These approaches demonstrate the value of machine vision for cotton-field monitoring and navigation, but they do not jointly delineate the pixel-level spatial distributions of crop rows, furrows, and obstacles within a unified label space. Semantic segmentation can provide this structured scene information and thereby support traversability assessment, navigation-path planning, and obstacle avoidance [6,7]. Nevertheless, accurate and computationally efficient multi-class segmentation remains challenging in seedling-stage cotton fields because of perspective-convergent crop rows, irregular furrow boundaries, and obstacles occupying only a small proportion of image pixels.

Early field-vision methods relied mainly on manually designed color, texture, and geometric features. [8] compared vegetation color indices under different soil, residue, and illumination conditions, providing classical features for separating vegetation from non-vegetated backgrounds. [9] combined vegetation enhancement, threshold segmentation, and line fitting to identify maize rows under high weed pressure, while [10] further distinguished crop rows from weeds using vegetation distributions and geometric relationships. These methods generally have low computational costs, but their performance depends strongly on handcrafted assumptions such as fixed color thresholds, nearly straight crop rows, and regular vegetation distributions. Consequently, they are susceptible to illumination changes, shadows, weeds, missing plants, and discontinuous rows. Deep learning has improved feature representation under complex conditions. DeepLabV3 + employs atrous convolution and an encoder–decoder architecture to strengthen multi-scale contextual modeling and boundary recovery [11], whereas SegFormer uses a hierarchical Transformer encoder to capture global semantic relationships [12]. For real-time segmentation, BiSeNetV2, DDRNet, STDC, and PIDNet use dual-branch processing, parallel multi-resolution representations, efficient feature aggregation, and proportional–integral–derivative-inspired branches, respectively, to balance segmentation accuracy and inference efficiency [13–16]. However, these general-purpose networks were not specifically designed for the agronomic and visual characteristics of seedling-stage cotton fields.

Recent studies have adapted semantic segmentation networks to specific agricultural perception tasks. For crop-row recognition and navigation, [17] incorporated atrous spatial pyramid pooling into U-Net and combined segmentation with region projection and line fitting to improve maize-row recognition under weed interference, discontinuous rows, and overlapping leaves. [18] developed a real-time segmentation network for crop-row navigation-line detection, whereas [19] combined deep-learning-based crop-row segmentation with visual servoing and evaluated robustness across varying field conditions. [20] proposed a lightweight segmentation method for oilseed rape row detection and robot navigation and evaluated transfer to other row-crop scenes. For resource-constrained crop and weed perception, [21] designed an attention-aided lightweight network intended for smart-weeding robot hardware. [22] used pixel-wise crop-row segmentation to extract navigation lines across sugarcane growth stages and assessed extension to maize and rice, while [23] developed CCRDNet for central crop-row extraction in universal in-row navigation. Beyond single-target crop-row perception, [24] integrated drivable-area segmentation and obstacle detection for orchard inter-row navigation, demonstrating the importance of combining traversability and safety information. Related work identifies robustness under complex field conditions, data diversity, cross-scene transferability, domain adaptation, and lightweight edge deployment as important directions for agricultural visual perception [25–27]. These studies demonstrate the value of task-specific model design. However, the existing tasks and label systems generally emphasize crop rows, weeds, navigation paths, or orchard drivable areas. Limited work has evaluated a unified pixel-level label space containing crop rows, furrows, and obstacles for real-time perception in seedling-stage cotton fields.

Accordingly, multi-class semantic segmentation in seedling-stage cotton fields involves three interrelated challenges. First, shallow fine-grained details are easily lost. Cotton seedlings, distant crop rows, and small-scale obstacles contain fragile edge and texture information that can be weakened by successive downsampling, resulting in discontinuous crop rows and missed obstacles. Second, deep global-structure modeling remains insufficient. Crop rows and furrows exhibit long-range extensions and perspective convergence, while local convolutions alone may not fully characterize row-wise dependencies under missing plants, discontinuous rows, and partial occlusion. Third, semantic–boundary fusion must be improved without imposing excessive computational costs. Crop-row edges, furrow transition regions, and obstacle contours are easily confused by weeds, plastic mulch, soil clods, and shadows, whereas simple pixel-wise fusion may not make full use of boundary information for local correction. In addition, models intended for mobile field platforms must maintain low parameter counts and computational costs and should retain a certain degree of adaptability across different crop scenes.

PIDNet-S was selected as the foundation architecture because its proportional (P), integral (I), and derivative (D) branches separately preserve spatial details, aggregate semantic context, and model boundary information. These functions correspond closely to the principal sources of segmentation error in seedling-stage cotton fields. Nevertheless, the original PIDNet-S does not explicitly enhance small-scale obstacle features in shallow layers, model long-range crop-row structures in deep layers, or refine locally ambiguous boundaries during final feature fusion. Therefore, this study investigates whether task-oriented enhancement of shallow details, global row structures, and boundary-guided fusion can improve multi-class field perception while retaining a lightweight real-time architecture.

To address these challenges, a four-class semantic segmentation dataset for seedling-stage cotton-field scenes was constructed, and Agri-PIDNet-S, a lightweight semantic segmentation network based on PIDNet-S, was developed. The network follows three complementary design objectives: shallow-detail enhancement, deep global row-structure modeling, and boundary-guided feature fusion. The main contributions of this study are summarized as follows:

1. A four-class pixel-wise annotated dataset of 709 seedling-stage cotton-field images was constructed. It jointly represents background, crop rows, furrows, and obstacles across varied viewing scales, crop densities, and field conditions within the study site, including weeds, shadows, damaged plastic mulch, and other interference.
2. Agri-PIDNet-S was developed from PIDNet-S using CA-MKIR for shallow-detail preservation, SaE-D2T for long-range row-structure modeling, and BWR-Bag for boundary-guided fusion. It achieved 92.37% mIoU and 88.67% obstacle IoU with 8.048 million parameters, 19.161 GFLOPs, and 155.67 FPS.
3. Comparative experiments, module- and component-level ablations, loss ablation, obstacle-scale analysis, and error analysis systematically evaluated the model. Zero-shot testing on 69 unseen tomato, watermelon, and maize images yielded 86.29% structural mIoU, providing preliminary evidence of cross-crop transfer.

## Materials and methods

### Dataset construction

To investigate the semantic segmentation of crop rows, furrows, and obstacles in seedling-stage cotton fields, a four-class pixel-wise annotated dataset was constructed from images acquired in real cotton-field environments. The images were captured using different handheld mobile devices and covered diverse viewing perspectives, object scales, and types of background interference. The class definitions were designed to address the visual perception requirements of agricultural machinery and field robots during inter-row operations, including crop arrangement, potentially traversable areas, and obstacles. All cotton images used for model development and internal evaluation originated from one farm in a single region during one seedling-stage acquisition period from May to June 2025. The dataset therefore supports evaluation under these acquisition conditions but was not designed to establish generalization across farms, regions, seasons, growth stages, lighting conditions, or acquisition platforms beyond those represented here.

#### Image acquisition and screening.

The images were acquired within two large cotton fields at Huaxing Farm in Changji Hui Autonomous Prefecture, Xinjiang Uygur Autonomous Region, China, at approximately 44.22° N and 87.30° E. Cotton is extensively cultivated in this area, where crop rows and furrows exhibit clear spatial regularity.

Image acquisition was conducted from May to June 2025 during the cotton seedling stage. At this stage, the canopy had not yet fully closed, and bare-soil furrows remained visible between adjacent crop rows. The scenes nevertheless included small and sparsely distributed plants, missing plants, discontinuous rows, weeds, shadows, damaged plastic mulch, and complex soil textures.

To diversify the imaging conditions, an iPhone 13 Pro, a realme GT, and a OnePlus Ace 2 Pro were used, yielding 648, 490, and 271 candidate images, respectively. The original image resolutions were 2448 × 2448, 3024 × 3024, 3072 × 3072, and 3456 × 3456 pixels. Natural handheld acquisition covered close-, medium-, and long-range views, different viewing directions and crop-row spacings, and field elements such as people, irrigation pipes, and monitoring equipment. Before annotation, the 1,409 candidate images were screened solely according to image quality and scene coverage, without reference to model predictions. Images with severe blur, defocus, abnormal exposure, extensive occlusion, indistinguishable crop-row or furrow structures, or near-duplicate viewpoints and content were removed, leaving 709 retained original images for annotation and model evaluation. Representative field scenes retained after screening are shown in Fig 1.

**Fig 1:**
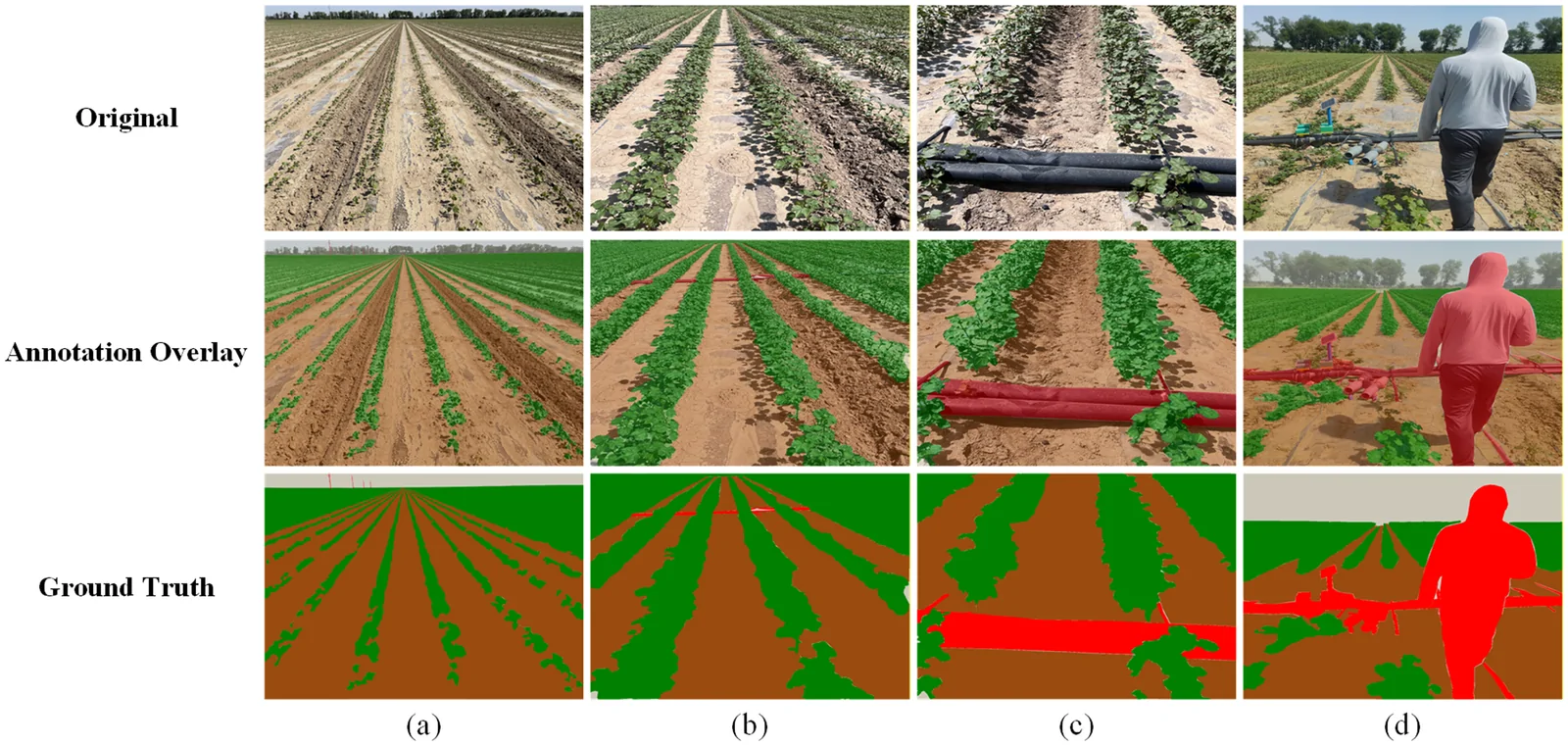
Representative seedling-stage cotton-field scenes and pixel-wise annotations. Columns (a)–(d) show (a) sparse crop rows with perspective convergence, (b) regular crop-row and furrow structures, (c) an irrigation-pipe obstacle, and (d) a person and nearby field facilities; the rows show the original images, annotation overlays, and ground-truth masks, respectively.

**Fieldwork permissions and ethics.** The main cotton-field site at Huaxing Farm was accessed under the established research collaboration between Xinjiang Agricultural University and Xinjiang Huaxing Investment Group Co., Ltd. The images were collected on experimental land made available for university research under this collaboration. Because the study involved only non-invasive photography within the agreed experimental site, no separate field-access permit or permit number was required. The site was agricultural land and was not designated as a protected area. No plants or animals were sampled, collected, or disturbed, and no protected species were involved. The research-team member depicted in Figs 1(d) and 9(d) provided written informed consent for publication of the images; the consent documentation is on file.

#### Semantic annotation and class distribution.

The dataset was annotated by two trained annotators with backgrounds in computer vision and agricultural engineering, respectively. Before annotation, the annotators and an agricultural engineering specialist agreed on the class definitions and boundary criteria. Each annotator checked their own annotations and subsequently cross-checked the annotations produced by the other annotator, after which the agricultural engineering specialist conducted a final semantic review and resolved disagreements.

The images were divided into four semantic classes: background, crop row, furrow, and obstacle. Polygonal pixel-wise annotations were created in LabelMe and converted into single-channel masks with labels 0–3. The labels represent navigation-relevant field structures rather than botanical categories. Crop rows represent cotton plants and continuous vegetation regions formed along the planting direction. Short bare-soil gaps and weeds located within a geometrically continuous planting row were assigned to the crop-row class, whereas inter-row soil, plastic mulch, and weeds were assigned to the furrow class. Visually identifiable people, irrigation facilities, monitoring equipment, and other objects that may obstruct agricultural vehicles were annotated as obstacles according to their visible contours without applying a fixed minimum pixel-area threshold; regions whose obstacle identity or boundary could not be determined reliably were not labeled as obstacles. All remaining regions were labeled as background. Masks were rasterized at the original image dimensions. During training, geometric transformations were applied synchronously to each image–mask pair, using nearest-neighbor interpolation for the mask. During validation and testing, the ground-truth masks remained at their original dimensions. Representative original images, annotation overlays, and ground-truth masks are shown in Fig 1.

To assess annotation quality, 70 images were sampled using stratification across the training, validation, and test subsets (49, 11, and 10 images, respectively) and across within-subset quartiles of obstacle area. An independent quality assessor who had participated in neither the initial annotation nor the preceding final semantic review inspected each original image, mask, and overlay without access to model predictions. The audit examined class assignment, missing or excessive labeled regions, visible boundary placement, obstacle labeling, and image–mask alignment. All 70 images met the predefined review criteria and required no label correction, and no systematic annotation problem was identified in the audited sample.

After annotation, image–mask correspondence, spatial dimensions, and pixel-value ranges were checked for consistency. Direct computation from all 709 original-resolution masks showed that background, crop rows, furrows, and obstacles accounted for 21.49%, 36.23%, 39.67%, and 2.60% of the pixels, respectively. Obstacle pixels appeared in 679 of the 709 images (95.77%) but generally occupied small regions, indicating high scene-level occurrence together with pronounced pixel-level class imbalance.

#### Dataset partitioning and augmentation.

Before partitioning, the 709 retained original images preserved their pre-existing numeric identifiers. Images belonging to the same continuous acquisition scene or file-number block were kept together as an indivisible unit and assigned to a single subset. Preserving group integrity was the primary allocation constraint, while obstacle content was considered only as a secondary factor when approaching the target subset sizes; identical obstacle-pixel distributions across subsets were not enforced. This group-aware allocation produced a fixed partition of 495 training, 107 validation, and 107 test images.

A retrospective audit of the fixed partition was conducted by establishing 86 acquisition/scene group identifiers from the preserved pre-split numeric image order and verified scene continuity. Every documented group occurred in exactly one subset. File-level and decoded-pixel SHA-256 checks identified no cross-subset exact duplicates. Perceptual-similarity screening followed by manual review identified no confirmed cross-subset near duplicates, and inspection of the augmentation workflow found no training-derived augmented image in the validation or test subset. These acquisition/scene groups document internal acquisition units within the two cotton fields and should not be interpreted as 86 physical plots.

The exact experimental partition is recorded in a fixed split manifest and does not require regeneration through a stochastic splitting procedure. Data augmentation was performed only after the fixed group-aware partition and only for the training subset.

Because the number of original training samples was limited, offline data augmentation was applied to expand the training set. Three augmented versions were generated once from each original training image and retained as a fixed offline-augmented set. The augmentation operations included brightness, contrast, and saturation perturbations; Gaussian blur; random rotations ranging from −15° to 15°; low-probability vertical flipping; and Gaussian noise. Different augmentation operations were combined according to predefined probabilities to increase variation in color appearance, image sharpness, geometry, and noise conditions.

Color perturbations, Gaussian blur, and Gaussian noise were applied only to the RGB images, whereas rotation and flipping were synchronously applied to the images and masks. Nearest-neighbor interpolation was used for the masks, and invalid boundary regions introduced by rotation were assigned an ignore label and excluded from loss calculation. Offline augmentation expanded the training set from 495 original images to 1,980 image–label pairs. The validation and test sets were not augmented and each retained 107 original images. Representative offline augmentation results are shown in Fig 2.

**Fig 2:**
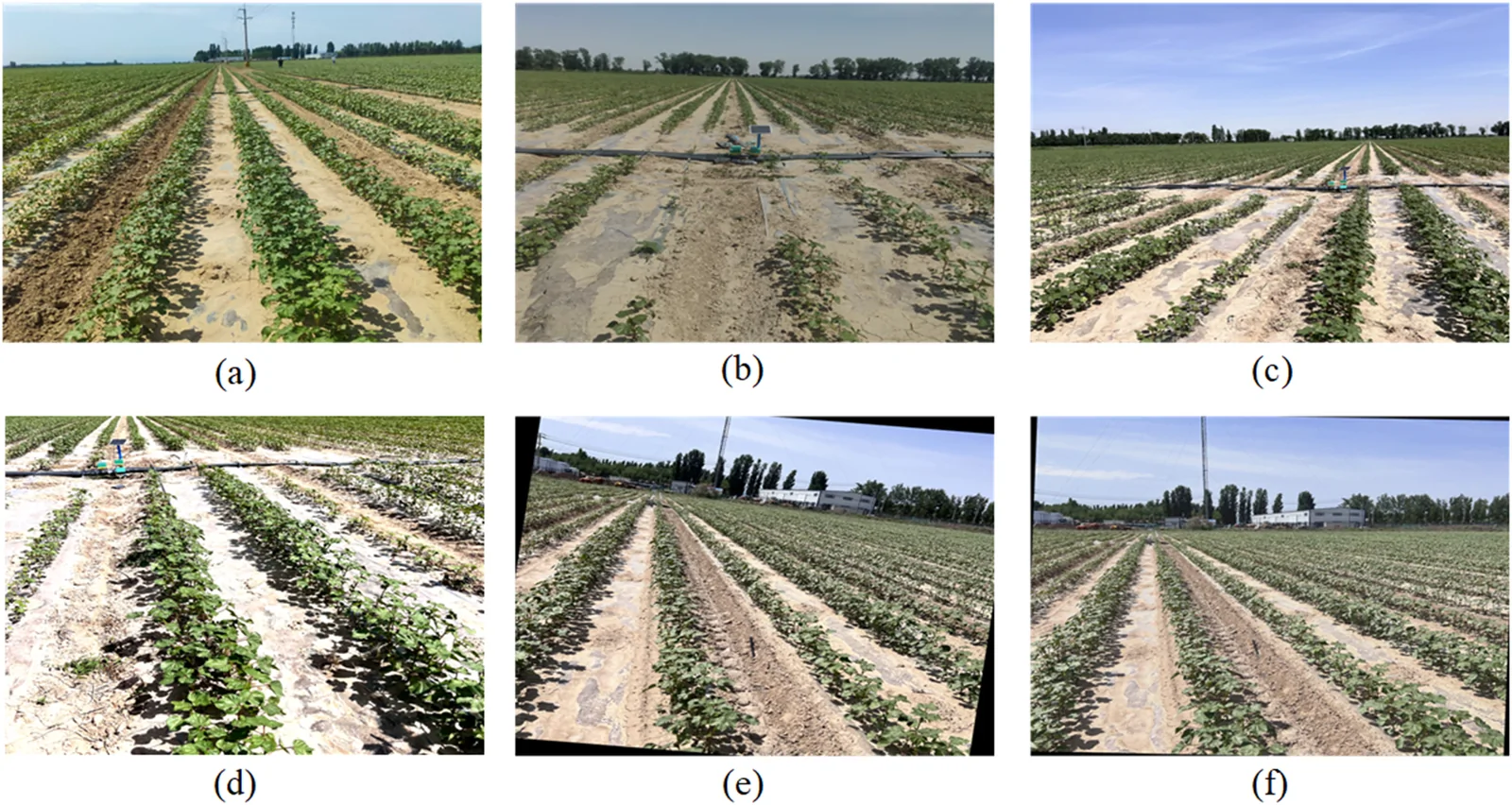
Representative examples of offline data augmentation applied to the training set. (a) Brightness, contrast, and saturation perturbations; (b) color perturbation and Gaussian blur; (c) Gaussian blur and Gaussian noise; (d) stronger color perturbation and Gaussian blur; (e) small-angle random rotation; and (f) small-angle rotation, color perturbation, and Gaussian noise.

During model training, online random augmentation was further applied to the offline-augmented training set. The input images were first preprocessed using a base scale of 2048 pixels, followed by random multi-scale resizing with scale factors ranging from 0.5 to 1.5, random cropping to 1024 × 1024 pixels, and random horizontal flipping. The images were then normalized using the ImageNet RGB mean and standard deviation reported in the “Experimental settings” subsection. Online random augmentation was applied only during training, whereas deterministic input-image resizing and normalization were used during validation and testing.

### Agri-PIDNet-S network

To address the characteristics of seedling-stage cotton-field scenes, including slender crop rows, continuous furrow structures, a low proportion of small-scale obstacles, and complex background interference, a lightweight semantic segmentation network named Agri-PIDNet-S was developed based on PIDNet-S [16]. The proposed network retains the fundamental design of the proportional branch (P branch), integral branch (I branch), and derivative branch (D branch) in PIDNet, which preserve spatial details, extract semantic context, and provide boundary-guidance information, respectively. To address the loss of shallow details, insufficient deep contextual modeling, and inadequate semantic–boundary fusion, three task-driven modules were introduced: a coordinate-aware multi-kernel inverted residual module (CA-MKIR), a squeeze-and-excitation-enhanced deep-to-token module (SaE-D2T), and a boundary-guided window-attention refinement Bag (BWR-Bag).

The PIDNet-S backbone used *m* = 2, *n* = 3, an initial width of 32 channels, 96 PAPPM channels, and 128 segmentation-head channels. The network input was 1024×1024 pixels, and the final head produced four semantic classes. These settings were retained as the architectural reference for all module variants.

#### Overall network architecture.

The overall architecture of Agri-PIDNet-S is illustrated in Fig 3. The input image is first processed by a shallow encoder to extract high-resolution initial features, which are subsequently delivered to three parallel branches: P, I, and D. The P branch maintains features at 1/8 of the input resolution to preserve local details, including crop-row edges, furrow boundaries, and obstacle contours. The I branch is progressively downsampled to 1/16, 1/32, and 1/64 of the input resolution to extract stronger semantic context and global row-wise structures. The D branch retains a relatively high spatial resolution to capture boundary variations and provide boundary guidance for the final feature fusion.

**Fig 3:**
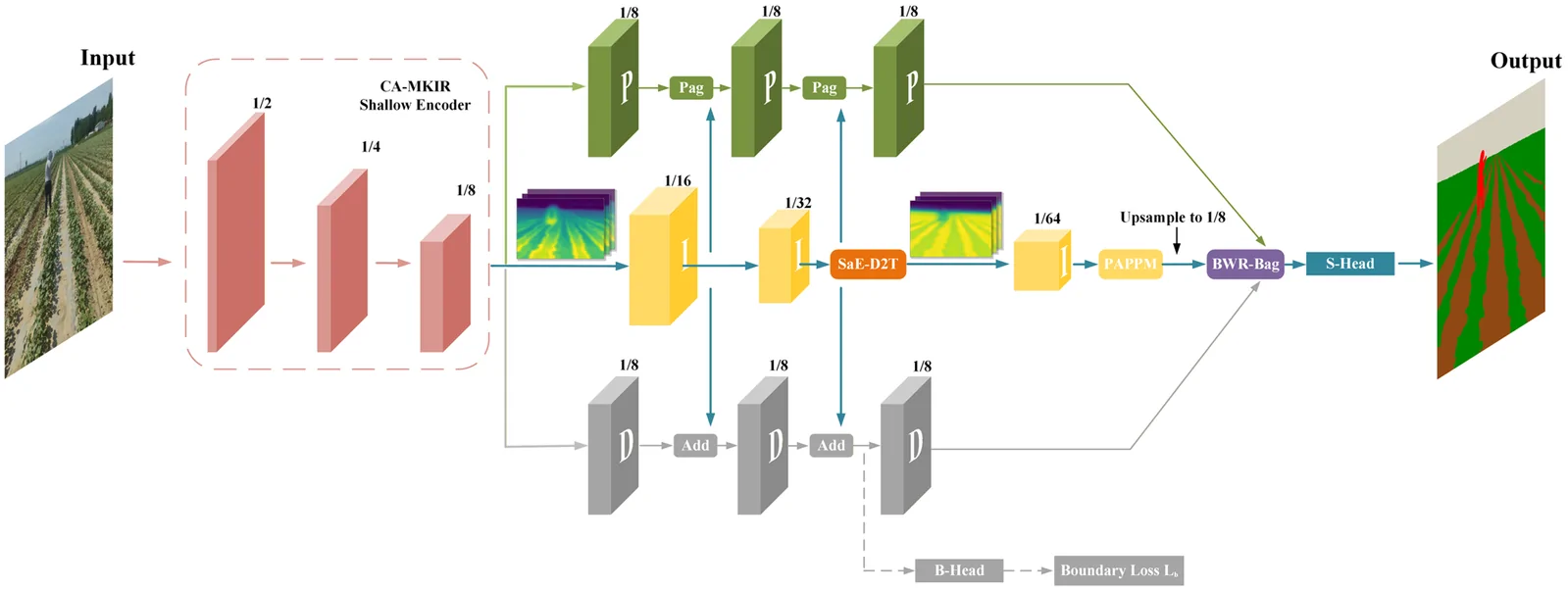
Overall architecture of Agri-PIDNet-S. The network consists of a shallow CA-MKIR encoder, the P-branch detail stream, the I-branch context stream, the D-branch boundary stream, and the BWR-Bag fusion module. Solid lines indicate the data flow during inference, whereas gray dashed lines indicate auxiliary boundary supervision applied during training.

In the I branch, the features are progressively downsampled to lower spatial resolutions as the network deepens. The SaE-D2T module is introduced at a deep stage of the I branch to enhance the global structural modeling of low-resolution semantic features. The features are then processed by the parallel aggregation pyramid pooling module (PAPPM) for multi-scale contextual aggregation and aligned to 1/8 of the input resolution through bilinear upsampling. Finally, BWR-Bag receives the high-resolution detail features from the P branch, the upsampled contextual features from the I branch, and the boundary features from the D branch. It fuses the three feature streams and produces the final semantic prediction.

#### Coordinate-aware multi-kernel inverted residual module.

In seedling-stage cotton-field images, cotton leaves are relatively small, while crop-row edges and furrow textures are primarily represented in shallow high-resolution features. The exclusive use of conventional convolutional residual blocks at shallow stages may result in insufficient representation of fine-grained information, such as leaf edges, soil cracks, and the contours of small-scale obstacles. Therefore, the CA-MKIR module was designed, as illustrated in Fig 4.

**Fig 4:**
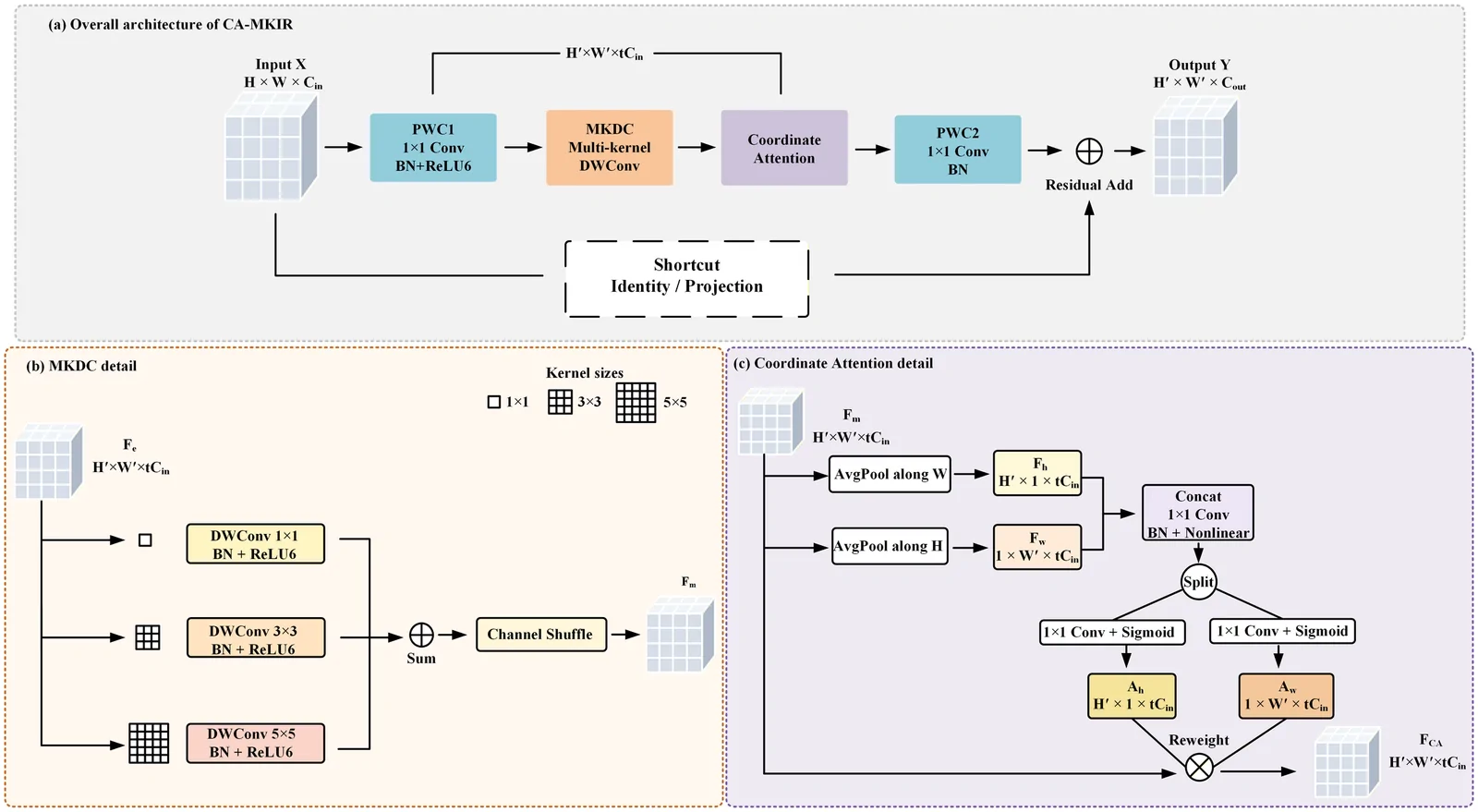
Architecture of the CA-MKIR module. (a) Overall module architecture; (b) details of the multi-kernel depthwise convolution; and (c) coordinate-attention refinement process.

In the implemented network, CA-MKIR used a channel-expansion ratio of *t* = 2 and replaced nine BasicBlocks: two blocks in layer1, two in layer2, two in the P-branch layer3_, two in the P-branch layer4_, and one in the D-branch layer3_d. Each replacement preserved the input and output dimensions of the corresponding PIDNet-S stage. The three depthwise branches used 1×1, 3×3, and 5×5 kernels with dilation 1.

As shown in Fig 4, CA-MKIR consists of pointwise channel expansion, multi-kernel depthwise convolution, channel shuffling, coordinate-attention refinement, and a residual connection [28–30]. Given an input feature $X\in {\mathbb{R}}^{H\times W\times C_{in}}$, PWC1, comprising a 1×1 pointwise convolution, batch normalization, and a ReLU6 activation function, is first applied for channel transformation, producing the expanded feature $F_{e}$. Subsequently, $F_{e}$ is fed into three depthwise convolution branches with kernel sizes of 1×1, 3×3, and 5×5. Each branch sequentially performs depthwise convolution, batch normalization, and ReLU6 activation using the same stride and output spatial dimensions, producing the multi-scale features *F*_1_, *F*_3_, and *F*_5_, respectively.

The three branches capture pointwise responses, local texture patterns, and broader local contextual information, respectively. The resulting multi-scale depthwise convolution features are aggregated through element-wise addition, followed by channel shuffling to enhance information interaction among branches with different receptive fields:


$$\begin{array}{c} F_{m}=\mathrm{Shuffle}\left(F_{1}+F_{3}+F_{5}\right), \end{array}$$ (1)

where $F_{m}$ denotes the aggregated multi-kernel feature, and Shuffle(·) denotes the channel-shuffling operation.

Subsequently, coordinate attention performs global average pooling along the width and height dimensions of the feature map, producing direction-aware encoded features of sizes $H^{\prime }\times 1\times tC_{in}$ and $1\times W^{\prime }\times tC_{in}$, respectively. Here, $H^{\prime }$ and $W^{\prime }$ denote the output feature height and width, respectively, *t* denotes the channel-expansion ratio, and *tC*_in_ denotes the channel dimension of the expanded feature $F_{e}$. The features from the two directions are concatenated, transformed by a shared convolu*t*ion, and then split. Each branch is further processed using a 1×1 convolution and a Sigmoid function to generate the height-direction attention map $A_{h}$ and width-direction attention map $A_{w}$. The coordinate-attention-refined feature is expressed as:


$$\begin{array}{c} F_{CA}=F_{m}⊙A_{h}⊙A_{w}, \end{array}$$ (2)

where ⊙ denotes element-wise multiplication with broadcasting. This operation enhances informative channel responses while preserving spatial positional information related to crop-row orientation, cotton-plant locations, and obstacle contours.

Finally, *F*_CA_ is processed by PWC2, which consists of a 1×1 convolution and batch normalization, to restore the required number of output channels. The resulting feature is then added to the shortcut connection to obtain the module output *Y*. When the input and output dimensions are identical, an identity mapping is used; otherwise, a projection mapping is applied to align the spatial resolution or number of channels. Through this design, CA-MKIR strengthens the shallow representations of cotton-plant edges, furrow textures, and small-scale obstacle contours.

#### Squeeze-and-excitation-enhanced deep-to-token module.

As the I branch progressively downsamples the feature maps, the deep features acquire stronger semantic representation capabilities, but their spatial resolution is substantially reduced. In seedling-stage cotton-field scenes, crop rows and furrows generally exhibit pronounced long-range continuity, while missing plants, discontinuous rows, perspective convergence, and partial occlusion further increase the difficulty of deep semantic understanding. Local convolutions alone may be insufficient to fully model long-range row-wise relationships. Therefore, the SaE-D2T module is introduced at a deep stage of the I branch to enhance the global structural perception of low-resolution semantic features.

D2T denotes Deep-to-Token, which converts deep low-resolution features into semantic tokens for global modeling. SaE denotes Squeeze-and-Excitation-enhanced, indicating that channel-response selection is further strengthened after semantic-token modeling [31]. To avoid confusion with the name of the overall module, the specific channel-enhancement unit is referred to as multi-branch channel recalibration (MBCR). The overall architecture of SaE-D2T is shown in Fig 5. The module primarily consists of sparse global attention, prototype semantic aggregation, and MBCR.

**Fig 5:**
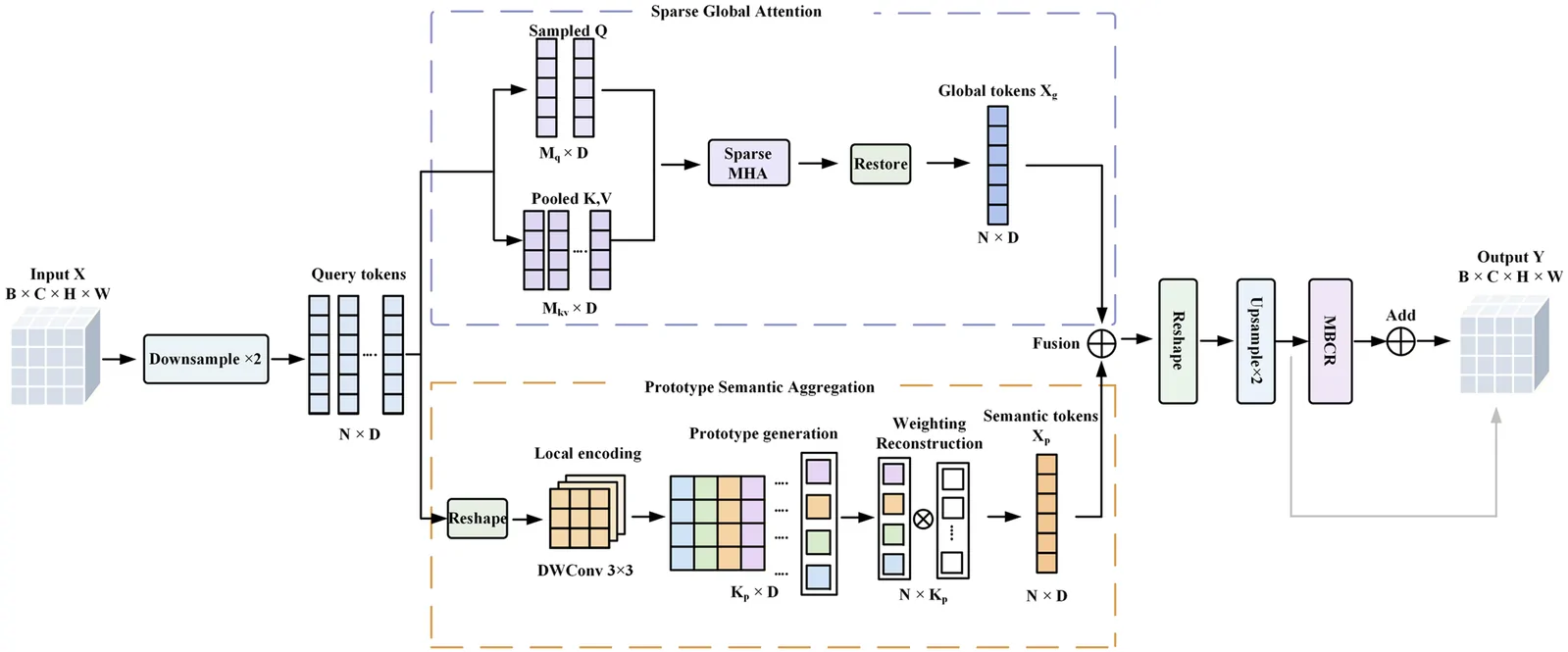
Architecture of the SaE-D2T module. The module consists of sparse global attention, prototype semantic aggregation, and multi-branch channel recalibration.

SaE-D2T does not replace PAPPM in the original PIDNet architecture. Instead, it is positioned before PAPPM to enhance global semantic interactions at the deep low-resolution stage, whereas PAPPM continues to perform multi-scale contextual aggregation. The two modules operate sequentially in the I branch and serve complementary roles in global semantic modeling and multi-scale contextual integration.

Given an input feature $X\in {\mathbb{R}}^{B\times C\times H\times W}$, where *B*, *C*, *H*, and *W* denote the batch size, number of channels, feature-map height, and feature-map width, respectively, average pooling is first applied to reduce the spatial resolution. The resulting feature is then flattened into query tokens $Q\in {\mathbb{R}}^{N\times D}$, where *N* denotes the number of tokens and *D* denotes the feature dimension of each token. The batch dimension is omitted from the token notation for simplicity. SaE-D2T contains two parallel paths, namely sparse global attention and prototype semantic aggregation, followed by channel recalibration using MBCR after feature fusion.

The pooled feature size was fixed at 8×8, yielding 64 tokens. Stride-4 sampling selected 16 query tokens (25% of the token sequence) for sparse attention, which used two effective attention heads. The prototype branch used $K_{p}=2$ prototypes. These prototypes were computed from the current input during each forward pass according to Eq. 5; they were not stored in a memory bank, updated through an exponential moving average, or represented as independent learnable prototype parameters.

To reduce the computational complexity of global attention while preserving long-range dependencies in deep features, a sparse global attention path is employed [32]. Fig 6 illustrates the detailed computational process of this path.

**Fig 6:**
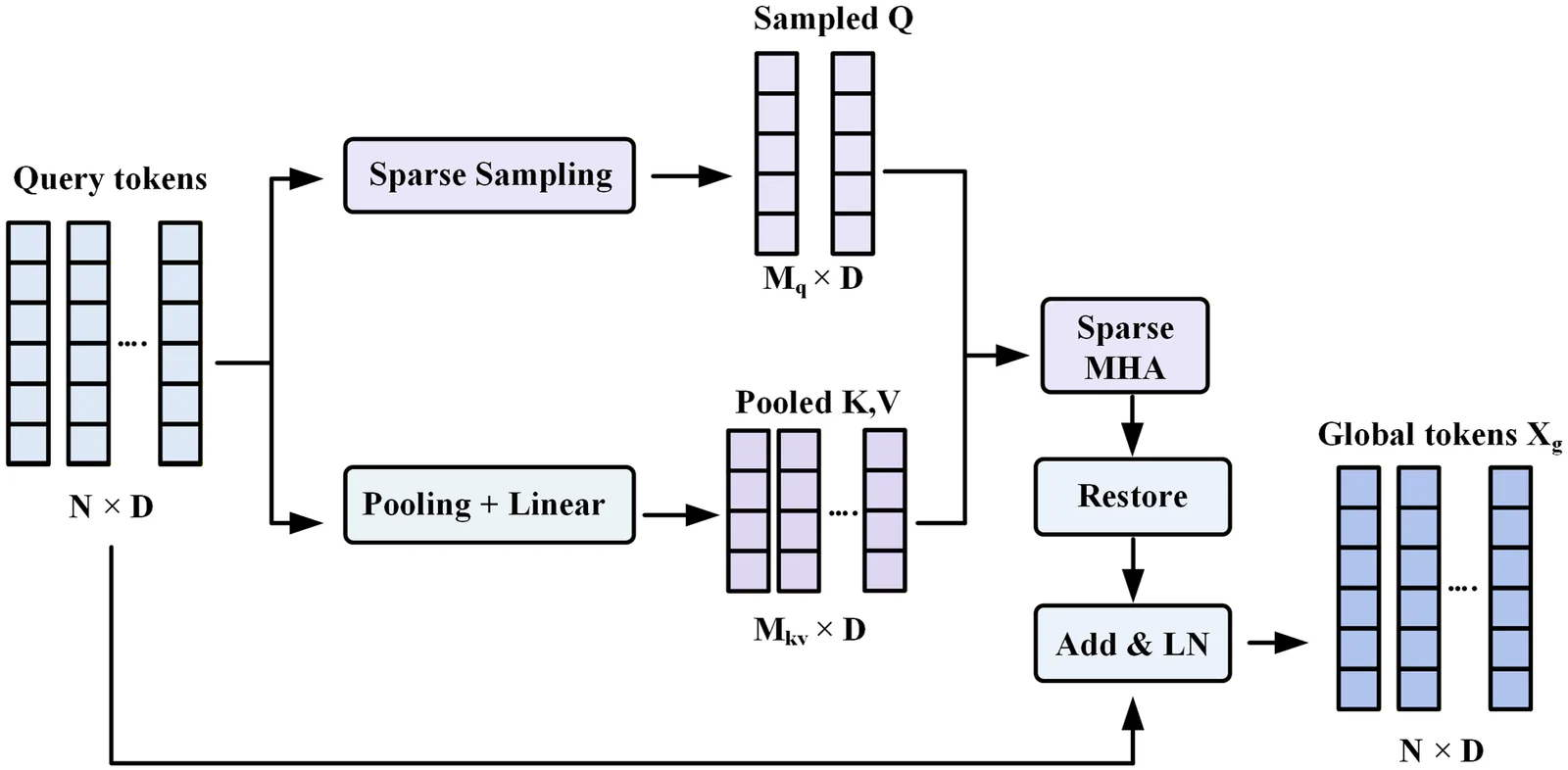
Sparse global attention path in SaE-D2T.

In this path, a subset of the query tokens is sampled to form $Q_{s}$, while $K_{s}$ and $V_{s}$ are generated through pooling and linear projection:


$$\begin{array}{c} Q_{s}=\mathrm{Sample}(Q),\;K_{s},V_{s}={𝒫}_{pool}(Q), \end{array}$$ (3)

The global semantic tokens are subsequently obtained through multi-head attention:


$$\begin{array}{c} X_{g}=\mathrm{LN}\;\left[Q+\mathrm{Restore}\;\left(\mathrm{MHA}(Q_{s},K_{s},V_{s})\right)\right], \end{array}$$ (4)

where Sample(·) denotes sparse token sampling, ${𝒫}_{pool}(\cdot )$ denotes pooling followed by linear projection, and MHA(·) denotes multi-head attention. Restore(·) restores the sparse-attention output to a complete token sequence of size N×D, while LN(·) denotes layer normalization. This path captures global dependencies among distant crop rows, thereby facilitating the processing of scenes involving perspective convergence, missing plants, discontinuous rows, and local interruptions in row structures.

In addition to modeling global dependencies, deep semantic features should be capable of structurally summarizing major regions, including crop rows, furrows, and the background. Therefore, a prototype semantic aggregation path is further incorporated into SaE-D2T. Fig 7 illustrates the computational process of this path.

**Fig 7:**
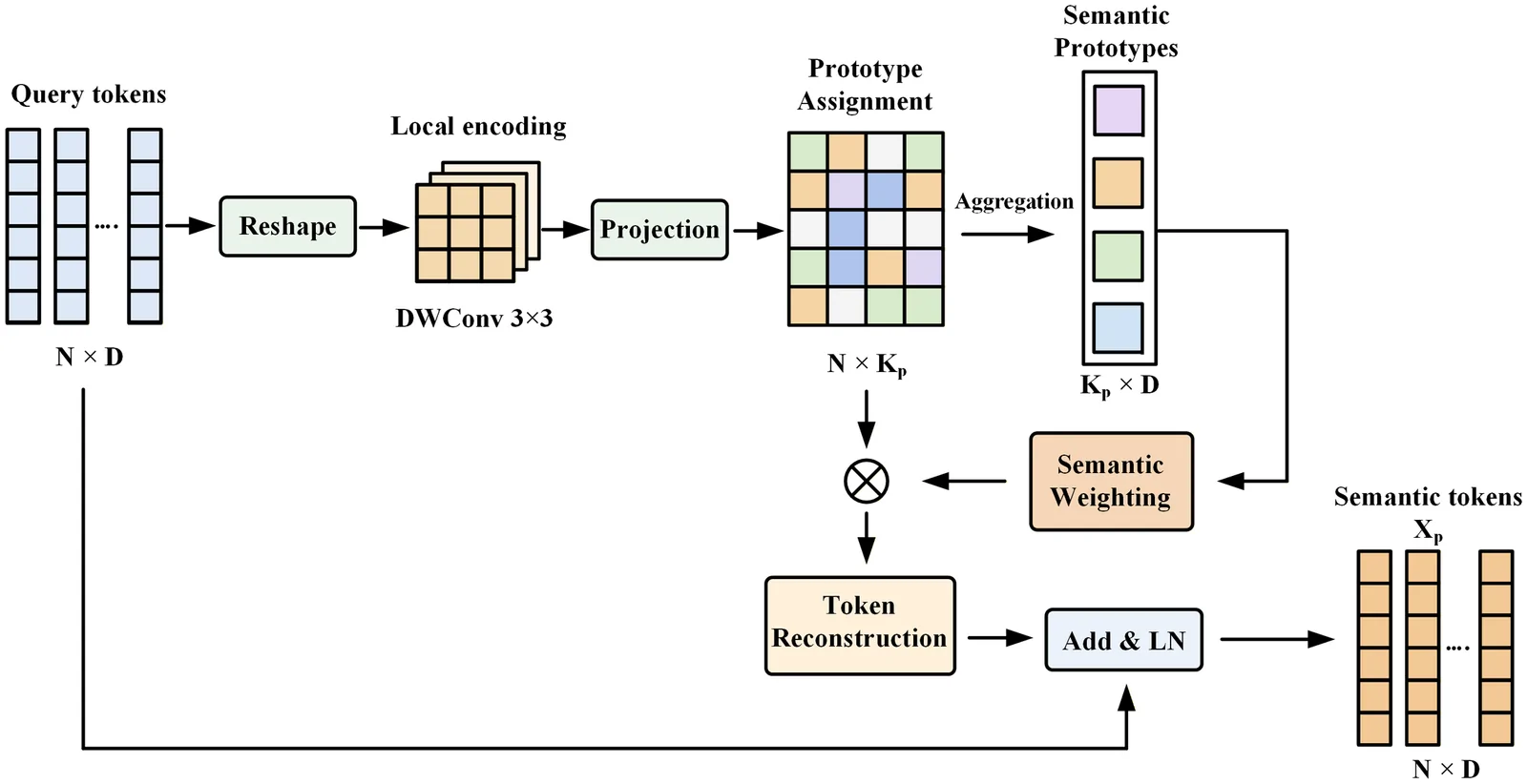
Prototype semantic aggregation path in SaE-D2T.

This path first obtains locally encoded tokens $\hat{Q}$ through a 3×3 depthwise convolution and linear projection, after which the normalized prototype-assignment weights $W_{p}$ are generated. The semantic prototypes and reconstructed tokens can be expressed as:


$$\begin{array}{c} P=W_{p}^{𝖳}\hat{Q},\;X_{p}=\mathrm{LN}\;\left[Q+W_{p}\Psi (P)\right], \end{array}$$ (5)

where $W_{p}\in {\mathbb{R}}^{N\times K_{p}}$ denotes the normalized assignment weights from the tokens to $K_{p}$ semantic prototypes, $P\in {\mathbb{R}}^{K_{p}\times D}$ denotes the aggregated semantic prototypes, Ψ(·) denotes the prototype feature transformation, and $X_{p}\in {\mathbb{R}}^{N\times D}$ denotes the prototype semantic tokens obtained after token reconstruction, residual addition, and layer normalization.

Subsequently, the sparse global tokens $X_{g}$ and prototype semantic tokens $X_{p}$ are fused and restored to a spatial feature map. To further enhance semantic selection across channels, MBCR is applied to the fused feature, and its output is expressed as:


$$\begin{array}{c} Y_{d2t}=R(X)+F_{deep}+F_{deep}⊙s, \end{array}$$ (6)

where *F*_deep_ denotes the fused and spatially restored deep semantic feature, *s* denotes the channel weights generated by MBCR, and *R*(*X*) denotes an identity or projection mapping of the input feature. Through the joint design of sparse global interaction, prototype semantic aggregation, and multi-branch channel recalibration, SaE-D2T improves the capacity of deep low-resolution features to model continuous crop-row structures, large-scale furrow layouts, and semantic differences in complex backgrounds.

MBCR operated on a 256-channel feature with a reduction ratio of 4 and a cardinality of 4. Global average pooling generated the channel descriptor, which was processed by four parallel channel-transformation branches. Each branch reduced the descriptor from *C* to *C*/4 channels; the four outputs were concatenated, projected back to *C* channels, and passed through a Sigmoid function to produce channel weights. The reweighted feature was combined with the residual path as expressed in Eq. 6.

#### Boundary-guided window-attention refinement Bag.

In PIDNet, the Bag module fuses the spatial-detail features from the P branch, semantic-context features from the I branch, and boundary features from the D branch [16]. Although this structure uses boundary information to adjust the fusion proportions of semantic and detail features, the original Bag module primarily relies on pixel-wise weighted fusion and has limited capacity to model local contextual relationships in the fused features. In seedling-stage cotton-field scenes, crop-row edges, furrow boundaries, and small-scale obstacle contours generally exhibit local continuity but are also susceptible to interference from weeds, soil clods, damaged plastic mulch, and illumination variations. Therefore, a single fusion operation based solely on boundary weights may be insufficient to fully recover continuous boundaries and slender structures.

To enhance boundary awareness and local structural refinement during the final fusion stage, the original Bag module is replaced with the boundary-guided window-attention refinement Bag. The architecture of BWR-Bag is illustrated in Fig 8. Drawing on the concepts of local window attention [33] and boundary-aware segmentation [34], the module takes the detail features from the P branch, contextual features from the I branch, and boundary features from the D branch as inputs. First, the D-branch features are used to generate a boundary-attention map that guides the adaptive fusion of the P- and I-branch features. Lightweight window attention is then applied to further refine the fused features.

**Fig 8:**
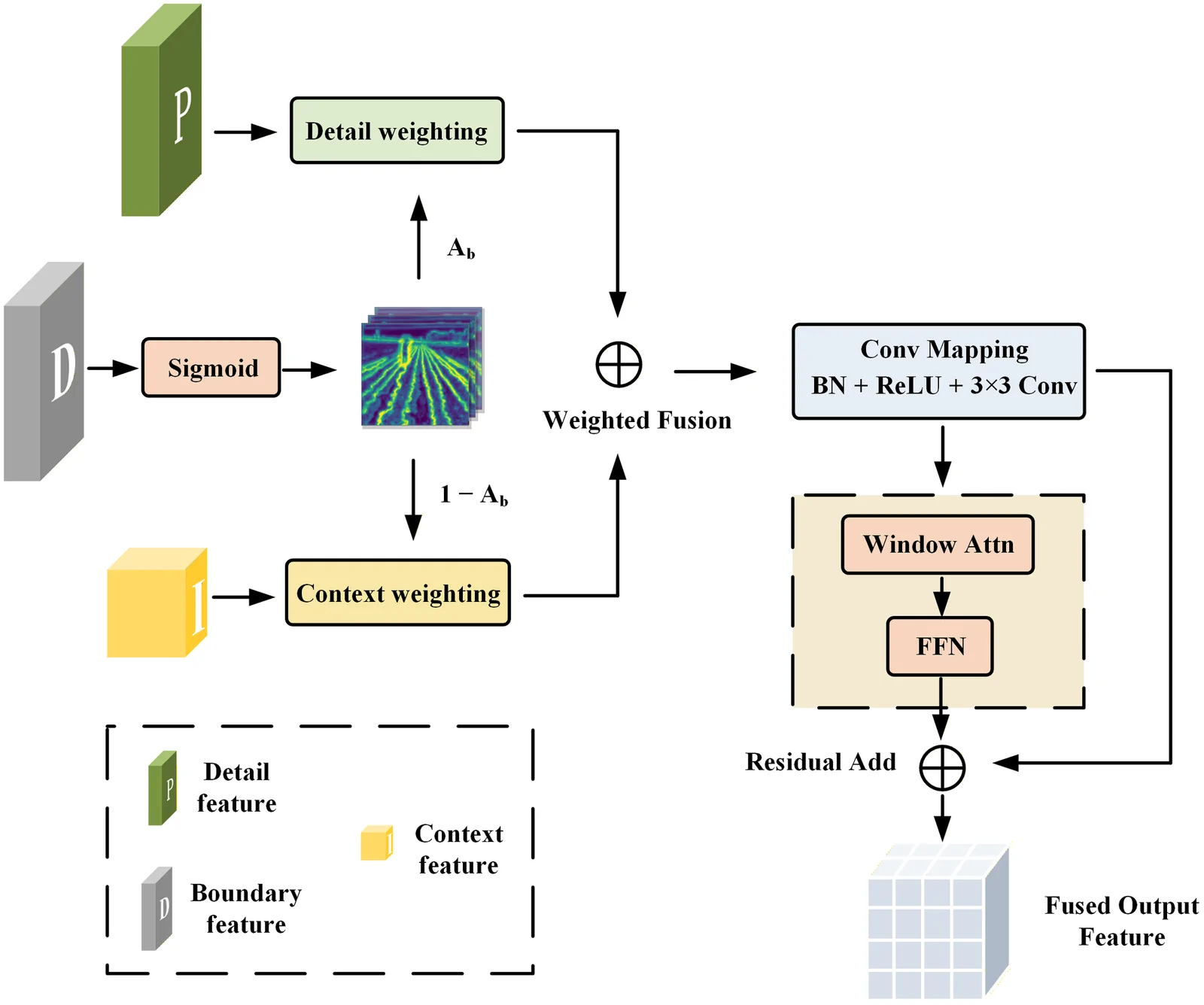
Architecture of BWR-Bag. The D branch generates a boundary-attention map to guide the weighted fusion of the P-branch detail features and I-branch contextual features, after which lightweight window attention further refines the fused features.

Given the P-branch detail feature $F_{p}$, I-branch contextual feature $F_{i}$, and D-branch boundary feature *F*_bd_, a Sigmoid mapping is first applied to *F*_bd_ to generate the boundary-attention map $A_{b}$. Meanwhile, the I-branch feature output by PAPPM is upsampled and, when necessary, channel-projected to match the spatial dimensions and number of channels of the P-branch feature. The aligned I-branch feature is denoted by $F_{i}^{\prime }$.

The boundary-attention map is used to regulate the fusion proportions of the detail and contextual features, and the fusion process is expressed as:


$$\begin{array}{c} F_{bag}=A_{b}⊙F_{p}+(1-A_{b})⊙F_{i}^{\prime }, \end{array}$$ (7)

where ⊙ denotes element-wise multiplication. When the boundary response is strong, the module places greater emphasis on the high-resolution local details from the P branch. Conversely, when the boundary response is weak, more semantic contextual information from the I branch is retained. In this manner, the D branch no longer serves solely as an independent boundary-prediction path but also participates in feature selection by providing boundary guidance during the final semantic fusion process. The weighted fused feature *F*_bag_ is subsequently processed by a feature-mapping operation consisting of batch normalization, ReLU activation, and a 3×3 convolution, producing the channel-aligned local feature $F_{c}$.

To further alleviate local boundary discontinuities and breaks in slender regions within the fused features, a lightweight window-attention refinement unit is introduced after $F_{c}$ [33]. Specifically, the fused feature $F_{c}$ is partitioned into non-overlapping local windows of size 4×4, within each of which self-attention and a feed-forward network (FFN) are applied. This local-window mechanism enhances structural consistency within neighboring regions while avoiding the high computational cost associated with global attention. To maintain the lightweight design of the network, no shifted-window mechanism is employed.

The BWR-Bag input and output features contained 128 channels. Window attention used two heads with 64 channels per head. Feature maps whose spatial dimensions were not divisible by four were padded by replication before window partitioning and restored to their original size afterward. Dropout was set to zero, and no relative-position bias was used.

Finally, the window-attention-refined feature is combined with the convolutionally mapped feature through a residual connection to obtain the output feature of BWR-Bag:


$$\begin{array}{c} Y_{bag}=F_{c}+W(F_{c}), \end{array}$$ (8)

where W(·) denotes the lightweight refinement operation composed of local-window self-attention and an FFN. The output feature *Y*_bag_ is subsequently passed to the final segmentation head to generate the semantic segmentation result. Through boundary-guided weighted fusion and window-attention refinement, BWR-Bag enhances the continuous representation of crop-row edges, furrow boundaries, and small-scale obstacle contours while maintaining a lightweight architecture, thereby improving the boundary integrity and structural consistency of the final segmentation results.

### Composite loss function

Seedling-stage cotton-field scenes exhibit pronounced class imbalance. Crop-row and furrow regions generally account for relatively large proportions of the image pixels, whereas obstacles, such as people, irrigation facilities, and field monitoring equipment, occupy considerably smaller pixel proportions and often have irregular boundaries. To improve the segmentation of small-scale obstacles, slender crop-row edges, and furrow boundaries, a composite loss incorporating auxiliary boundary supervision is adopted.

The overall loss function of Agri-PIDNet-S is defined as:


$$\begin{array}{c} L_{total}=\lambda_{ce}L_{ce}+\lambda_{dice}L_{dice}+\lambda_{lovasz}L_{lovasz}+\lambda_{b}L_{b}, \end{array}$$ (9)

where *L*_ce_, *L*_dice_, and *L*_lovasz_ denote the cross-entropy, Dice, and Lovász losses, respectively, and $L_{b}$ denotes the auxiliary boundary loss applied to the D branch. The corresponding balancing coefficients are denoted by $\lambda_{ce}$, $\lambda_{dice}$, $\lambda_{lovasz}$, and $\lambda_{b}$. In this study, these coefficients were set to $\lambda_{ce}=0.4$, $\lambda_{dice}=0.25$, $\lambda_{lovasz}=0.15$, and $\lambda_{b}=0.20$. The coefficients were empirically determined through preliminary experiments by considering differences in the magnitudes and relative importance of the individual loss terms. Their sum was constrained to one to maintain a stable overall scale of the composite loss. The semantic segmentation losses optimize the final pixel-level classification results, whereas the auxiliary boundary loss constrains the D branch to learn structural information, including crop-row edges, furrow boundaries, and obstacle contours. The cross-entropy loss provides stable pixel-level semantic classification supervision and is defined as:


$$\begin{array}{c} L_{ce}=-\frac{1}{N}\sum_{n=1}^{N}\sum_{c=1}^{C}y_{n,c}\log(p_{n,c}), \end{array}$$ (10)

where *N* denotes the number of pixels included in the calculation, *C* denotes the number of semantic classes, $y_{n,c}$ is the ground-truth indicator for pixel *n* belonging to class *c*, and $p_{n,c}$ is the probability that the model assigns pixel *n* to class *c*. The Dice loss measures the overlap between the predicted and ground-truth regions and helps alleviate class imbalance, particularly for classes with low pixel proportions, such as obstacles [35]. For the multi-class semantic segmentation task, the Dice loss is defined as:


$$\begin{array}{c} L_{dice}=1-\frac{1}{C}\sum_{c=1}^{C}\frac{2\sum_{n=1}^{N}p_{n,c}y_{n,c}+\varepsilon }{\sum_{n=1}^{N}p_{n,c}+\sum_{n=1}^{N}y_{n,c}+\varepsilon }, \end{array}$$ (11)

where *N* denotes the number of valid pixels included in the loss calculation, and *C* denotes the number of semantic classes, with *C* = 4 in this study. $p_{n,c}$ denotes the probability that the model assigns pixel *n* to class *c*, $y_{n,c}$ denotes the binary indicator of whether pixel *n* belongs to class *c*, and ε is a smoothing term used to prevent division by zero and improve numerical stability. The final Dice loss is obtained by taking the arithmetic mean of the class-specific Dice losses over the four semantic classes.

The Lovász loss optimizes an objective directly related to IoU, thereby improving the consistency between the training objective and the semantic segmentation evaluation metric [36]:


$$\begin{array}{c} L_{lovasz}=\frac{1}{C}\sum_{c=1}^{C}{\overset{―}{\Delta }}_{J_{c}}\;\left(m(c)\right), \end{array}$$ (12)

where ${\overset{―}{\Delta }}_{J_{c}}$ denotes the Lovász convex extension of the Jaccard loss for class *c*, and *m*(*c*) denotes the pixel-wise prediction-error vector for class *c*. The Lovász loss sorts the elements of *m*(*c*) and computes their weighted sum using the discrete gradient of the Jaccard loss, thereby approximating the optimization of an IoU-related objective.

To encourage the D branch to learn more explicit boundary responses, auxiliary boundary supervision is introduced during training. Following boundary-branch supervision used in boundary-aware segmentation networks [16,34], boundary pixels were identified from differences between adjacent labels in the semantic mask using the MMSegmentation GenerateEdge transform, after which the binary edge map was dilated with edge_width = 4. The resulting labels guide the D branch toward crop-row edges, furrow boundaries, and obstacle contours. The auxiliary boundary loss adopts the unweighted binary cross-entropy form:


$$\begin{array}{c} L_{b}=-\frac{1}{N}\sum_{n=1}^{N}\left[g_{n}\log(q_{n})+(1-g_{n})\log(1-q_{n})\right], \end{array}$$ (13)

where $g_{n}$ denotes the boundary label of pixel *n*, and $q_{n}$ denotes the boundary probability predicted by the D branch. After training, the D branch is not evaluated as an independent output; instead, it serves as a boundary-feature stream that participates in feature fusion within BWR-Bag.

### Experimental settings

All experiments were conducted on Ubuntu 22.04 using Python 3.10, PyTorch 2.1.0 + cu121, CUDA 12.1, cuDNN 8.9, MMEngine 0.10.3, MMCV 2.1.0, and MMSegmentation 1.2.2. Training and inference were performed on a single NVIDIA GeForce RTX 4090 GPU with 24 GB of memory.

The data preprocessing and augmentation procedures followed the “Dataset partitioning and augmentation” subsection. During training, 1024×1024 image–mask patches generated through random multi-scale resizing, random cropping, horizontal flipping, and normalization were used as network inputs. For validation and testing, BGR images read by MMSegmentation were converted to RGB, resized directly to 1024×1024 pixels using bilinear interpolation without padding, and normalized using RGB means [123.675, 116.280, 103.530] and standard deviations [58.395, 57.120, 57.375]. The ground-truth masks were loaded and retained at their original image dimensions; model predictions were restored to the corresponding original dimensions before metric calculation. To ensure a fair comparison, all models used the same dataset split, input size, training protocol, and evaluation procedure.

All models were trained using the AdamW optimizer [37] with an initial learning rate of $5\times 10^{-4}$ and a weight decay of 0.01. The learning rate followed a polynomial decay schedule with a power of 0.9, preceded by a linear warm-up over the first 1,500 iterations. Training lasted 20,000 iterations with a batch size of 4. Gradient clipping with a maximum norm of 1.0 was applied to improve training stability. For the model-comparison and ablation experiments, the checkpoint saved at the final 20,000th iteration was used for evaluation.

To assess training stochasticity, the complete Agri-PIDNet-S model was independently trained using seeds 0, 1, and 3407. All three runs used the same fixed 495/107/107 partition and the same set of 1,980 offline-augmented training image–mask pairs. The architecture, pretrained initialization protocol, loss functions, optimizer, learning-rate schedule, training duration, and checkpoint-selection rule were unchanged. Only seed-controlled model initialization, data shuffling, and online stochastic augmentation varied. Results are summarized using the individual runs and the mean ± sample standard deviation (SD). The model-comparison and obstacle-metric results report the seed-0 run so that all models are presented under the same single-run comparison protocol.

Computational efficiency was measured on the same RTX 4090 using batch size 1, FP32 arithmetic, and 1024×1024 inputs. FPS represents model-forward throughput only: five warm-up passes were followed by 195 timed passes from a total of 200 runs, excluding image loading, preprocessing, CPU-to-GPU transfer, and post-processing. FLOPs were obtained using the MMEngine counter under the convention that one multiply–accumulate operation was counted as one operation.

### Evaluation metrics

To comprehensively evaluate model performance on the semantic segmentation task in seedling-stage cotton fields, the evaluation was conducted from three perspectives: segmentation accuracy, obstacle-recognition performance, and computational efficiency. The segmentation accuracy metrics included the intersection over union (IoU) for the background, crop-row, furrow, and obstacle classes, mean intersection over union (mIoU), and mean Dice coefficient (mDice). Because obstacles account for a relatively small proportion of the dataset pixels but are critical to field-navigation safety, obstacle precision, obstacle recall, and obstacle F1-score were additionally reported. Computational efficiency was evaluated using the number of parameters (Params), floating-point operations (FLOPs), and inference speed in frames per second (FPS).

For class *c*, IoU is defined as:


$$\begin{array}{c} {\mathrm{IoU}}_{c}=\frac{{TP}_{c}}{{TP}_{c}+{FP}_{c}+{FN}_{c}}, \end{array}$$ (14)

where c∈{1,...,C} denotes the class index, *C* denotes the number of semantic classes, and *C* = 4 in this study. ${TP}_{c}$, ${FP}_{c}$, and ${FN}_{c}$ denote the numbers of true-positive, false-positive, and false-negative pixels for class *c*, respectively. The mean IoU over all classes is defined as:


$$\begin{array}{c} \mathrm{mIoU}=\frac{1}{C}\sum_{c=1}^{C}{\mathrm{IoU}}_{c}. \end{array}$$ (15)

The Dice coefficient measures the overlap between the predicted and ground-truth regions and is defined as:


$$\begin{array}{c} {\mathrm{Dice}}_{c}=\frac{2{TP}_{c}}{2{TP}_{c}+{FP}_{c}+{FN}_{c}}. \end{array}$$ (16)

The mean Dice coefficient over all classes is defined as:


$$\begin{array}{c} \mathrm{mDice}=\frac{1}{C}\sum_{c=1}^{C}{\mathrm{Dice}}_{c}. \end{array}$$ (17)

Because obstacle pixels account for a relatively small proportion of the dataset and are closely related to field-operation safety, precision, recall, and F1-score were additionally reported for the obstacle class. Obstacle precision represents the proportion of correctly predicted obstacle pixels among all pixels predicted as obstacles and reflects the reliability of the obstacle predictions. Obstacle recall represents the proportion of correctly identified obstacle pixels among all ground-truth obstacle pixels and reflects the model’s ability to identify obstacles. The obstacle F1-score is the harmonic mean of precision and recall and provides a combined assessment of the accuracy and completeness of obstacle recognition.

In this study, mIoU was used as the primary metric for overall segmentation accuracy. Obstacle IoU, precision, recall, and F1-score were used as supplementary metrics for evaluating obstacle-perception performance. Params, FLOPs, and FPS were also considered to analyze the balance between segmentation accuracy and real-time inference performance.

For the cross-crop external evaluation, image-level 95% confidence intervals (CIs) were estimated using a nonparametric cluster bootstrap with 10,000 resamples and statistical seed 20260917. Each crop-specific bootstrap sampled complete images with replacement from the corresponding crop subset, retaining the original subset size; the Overall bootstrap sampled 69 images with replacement from the pooled set without stratification by crop. The pixel confusion matrices of the sampled images were aggregated before recalculating each metric. Metrics with a zero denominator were recorded as undefined and excluded only from the percentile calculation for that metric. Obstacle F1-score was calculated directly as 2TP/(2TP + FP + FN), so a resample containing no ground-truth obstacle pixels but nonzero false-positive obstacle predictions contributed an F1-score of zero. The 95% CIs correspond to the 2.5th and 97.5th percentiles of the bootstrap distribution.

## Results

### Comparison with representative semantic segmentation networks

To verify the effectiveness of Agri-PIDNet-S for semantic segmentation in seedling-stage cotton-field scenes, DeepLabV3 + , DDRNet-23-slim, SegFormer-B0, BiSeNetV2, STDC2-Seg, PIDNet-S, and PIDNet-M were selected as comparison models [11–16]. All models were trained and evaluated using the same dataset split, input resolution, training protocol, and evaluation procedure to ensure a fair comparison. The class-wise IoU, mIoU, mDice, and computational-efficiency metrics of the different models on the test set are presented in Table 1.

**Table 1:** Performance comparison of different segmentation models.

Model	Background IoU (%)	Crop row IoU (%)	Furrow IoU (%)	Obstacle IoU (%)	mIoU (%)	mDice (%)	Params (M)	FLOPs (G)	FPS
DeepLabV3+ (MobileNetV2)	89.67	89.02	88.16	70.75	84.40	91.33	5.220	67.390	101.60
DDRNet-23-slim	86.02	90.87	91.48	78.60	86.74	92.82	5.700	36.640	183.60
SegFormer-B0	85.98	89.64	90.08	71.88	84.40	91.35	3.720	87.440	76.70
BiSeNetV2	83.87	89.88	89.83	77.16	85.19	91.91	**3.340**	49.410	**230.34**
STDC2-Seg	86.14	91.05	91.60	78.13	86.73	92.80	16.070	146.940	135.00
PIDNet-S	91.88	92.44	91.43	79.60	88.84	94.00	7.624	23.830	140.17
PIDNet-M	92.40	93.05	92.15	81.70	89.83	94.58	28.537	89.160	61.85
**Agri-PIDNet-S**	**93.31**	**94.06**	**93.45**	**88.67**	**92.37**	**96.02**	8.048	**19.161**	155.67

*Note:* Bold values indicate the best result in each metric column. FLOPs were calculated using a 1024×1024 input. FPS denotes batch-1 inference throughput measured on a single NVIDIA GeForce RTX 4090 under identical software and hardware settings.

Under the common single-run protocol shown in Table 1, Agri-PIDNet-S produced the highest point estimates for overall segmentation performance, with an mIoU of 92.37% and an mDice of 96.02%. Compared with the original PIDNet-S, Agri-PIDNet-S improved mIoU and mDice by 3.53 and 2.02 percentage points, respectively. In particular, the obstacle IoU increased from 79.60% to 88.67%, representing an improvement of 9.07 percentage points. These observed differences are consistent with improved segmentation of small-scale obstacles, furrow boundaries, and crop-row details under the evaluated protocol.

Compared with the larger PIDNet-M, Agri-PIDNet-S improved mIoU, mDice, and obstacle IoU by 2.54, 1.44, and 6.97 percentage points, respectively. Meanwhile, Agri-PIDNet-S contained 8.048 million parameters, compared with 28.537 million for PIDNet-M, and its computational cost was 19.161 rather than 89.16 GFLOPs. Its inference speed reached 155.67 FPS, compared with 61.85 FPS for PIDNet-M, corresponding to approximately 2.52 times the throughput under the same evaluation protocol. Under this single-run comparison, Agri-PIDNet-S therefore achieved higher segmentation scores than PIDNet-M with markedly lower model capacity.

Regarding class-wise performance, Agri-PIDNet-S achieved IoU values of 93.31%, 94.06%, 93.45%, and 88.67% for the background, crop-row, furrow, and obstacle classes, respectively. The crop-row and furrow results are consistent with improved preservation of slender crop rows and bare-soil furrow regions under the evaluated protocol. The largest increase over PIDNet-S occurred for the obstacle class, for which IoU increased by 9.07 percentage points.

In terms of computational efficiency, Agri-PIDNet-S required 19.161 GFLOPs, the lowest among all compared models, and achieved an inference speed of 155.67 FPS. Although BiSeNetV2 and DDRNet-23-slim achieved higher inference speeds, their mIoU and obstacle IoU values were substantially lower than those of Agri-PIDNet-S. Compared with DeepLabV3 + , SegFormer-B0, STDC2-Seg, and PIDNet-M, Agri-PIDNet-S attained higher segmentation accuracy with markedly lower computational complexity. It therefore provides a more favorable accuracy–efficiency trade-off for real-time visual perception in seedling-stage cotton fields.

Although the parameter count increased only slightly from 7.624 million to 8.048 million, the computational cost decreased from 23.83 to 19.161 GFLOPs, while the inference speed increased from 140.17 to 155.67 FPS. This is because CA-MKIR replaces selected standard convolutional residual blocks with depthwise convolutions and inverted-residual operations, while BWR-Bag replaces the original Bag module rather than being appended to it. Consequently, the additional semantic-modeling components slightly increase the model size, whereas computation in the high-resolution stages is reduced.

Because obstacle pixels account for only a small proportion of the dataset but are important for environmental perception in field navigation, the precision, recall, and F1-score of each model for the obstacle class were further evaluated, as reported in Table 2.

**Table 2:** Obstacle-class precision, recall, and F1-score for different models.

Model	Obstacle precision (%)	Obstacle recall (%)	Obstacle F1-score (%)
DeepLabV3+ (MobileNetV2)	73.24	95.43	82.87
DDRNet-23-slim	79.94	**97.90**	88.02
SegFormer-B0	73.03	97.85	83.64
BiSeNetV2	79.55	96.26	87.11
STDC2-Seg	79.51	97.84	87.72
PIDNet-S	89.49	87.80	88.64
PIDNet-M	90.75	89.12	89.93
**Agri-PIDNet-S**	**93.89**	94.09	**93.99**

*Note:* Bold values indicate the best result in each metric column.

As shown in Table 2, Agri-PIDNet-S achieved obstacle precision, recall, and F1-score values of 93.89%, 94.09%, and 93.99%, respectively. Five comparison models achieved higher obstacle recall values, ranging from 95.43% to 97.90%, but their precision values were lower, ranging from 73.03% to 79.94%. The closely matched precision and recall of Agri-PIDNet-S indicate a balanced obstacle-segmentation result.

To assess run-to-run variability of the complete model, the overall and class-level results from the three training seeds are summarized in Table 3.

**Table 3:** Training-seed stability of the complete Agri-PIDNet-S model. Values are percentages; class-level entries are the mean ± sample SD across the three runs.

Overall performance by training seed				
Training seed	mIoU (%)		mDice (%)	
0	92.37		96.02	
1	92.32		95.99	
3407	92.35		96.01	
Mean ± SD	92.35 ± 0.03		96.01 ± 0.02	
**Class-level performance across the three seeds**				
**Class**	**IoU (%)**	**Precision (%)**	**Recall (%)**	**F1-score (%)**
Background	93.35 ± 0.10	98.91 ± 0.08	94.32 ± 0.06	96.56 ± 0.05
Crop row	94.03 ± 0.03	96.57 ± 0.04	97.27 ± 0.05	96.92 ± 0.02
Furrow	93.46 ± 0.03	95.73 ± 0.06	97.52 ± 0.06	96.62 ± 0.02
Obstacle	88.54 ± 0.14	94.01 ± 0.14	93.83 ± 0.23	93.92 ± 0.08

*Note:* Seed 0 is the run reported in Tables 1 and 2.

As shown in Table 3, the mIoU values for seeds 0, 1, and 3407 were 92.37%, 92.32%, and 92.35%, respectively, yielding 92.35 ± 0.03%; the maximum difference was 0.05 percentage points. The corresponding mDice was 96.01 ± 0.02%, and the obstacle IoU was 88.54 ± 0.14%. These small variations indicate low sensitivity of the reported Agri-PIDNet-S performance to the three tested training seeds under the fixed experimental protocol. The repeated runs characterize the training variability of Agri-PIDNet-S, whereas the cross-model differences in Tables 1 and 2 remain point-estimate comparisons.

To provide a more intuitive comparison of the segmentation results produced by different models, Fig 9 presents predictions for representative seedling-stage cotton-field images. Fig 9(a) shows a scene with regular crop rows and clearly defined furrow structures, allowing comparison of the models’ ability to preserve crop-row continuity and furrow boundaries. Fig 9(b) presents crop-row segmentation under long-range perspective convergence and evaluates the recognition of narrow distal crop rows and inter-row structures. Fig 9(c) contains irrigation-facility interference, distant small-scale obstacles, and variations in inter-row structure, thereby illustrating model behavior in a complex field-facility environment. Fig 9(d) shows a challenging scene containing a person and nearby field facilities and is used to examine the delineation of large obstacle boundaries and surrounding regions. Collectively, these samples cover common challenges in seedling-stage cotton-field perception, including crop-row continuity, furrow-boundary delineation, distant small-object segmentation, person recognition, and complex background interference.

**Fig 9:**
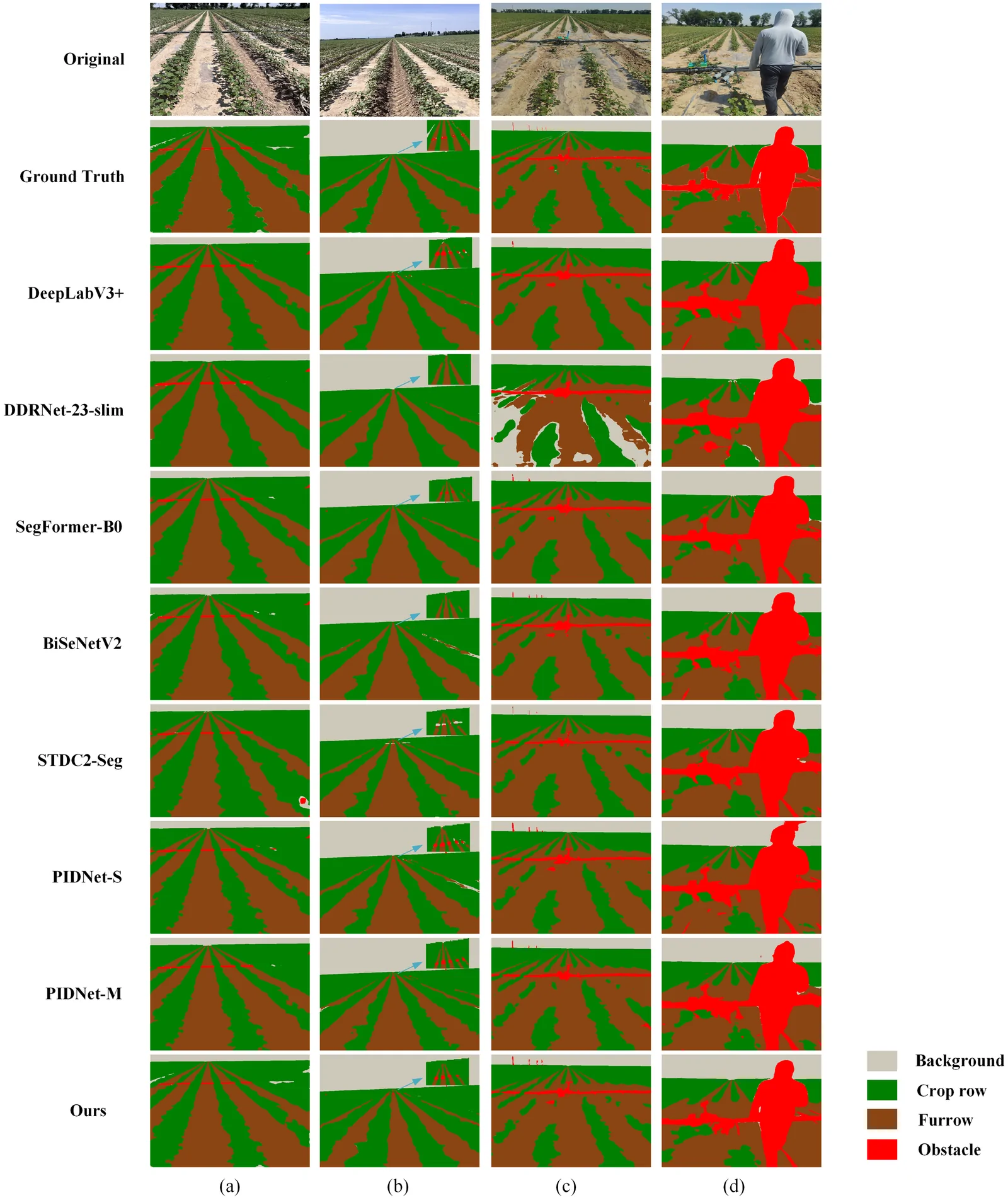
Visual comparison of predictions produced by different semantic segmentation models in representative seedling-stage cotton-field scenes. (a) Regular crop-row and furrow structures; (b) long-range perspective convergence; (c) irrigation-facility interference and distant small-scale objects; and (d) a person and interference from nearby field facilities.

### Ablation study of the proposed modules

To evaluate both the individual and joint contributions of CA-MKIR, SaE-D2T, and BWR-Bag, a full 2^3^ factorial ablation was conducted using the original PIDNet-S as the baseline. All eight possible module combinations were trained and evaluated using seed 0, the same fixed data partition and offline-augmented training set, the same pretrained initialization protocol, training schedule, checkpoint-selection rule, test set, and evaluation procedure. The class-wise IoU, mIoU, and mDice results are reported in Table 4.

**Table 4:** Full-factorial ablation results for the proposed modules in Agri-PIDNet-S.

Variant	CA-MKIR	SaE-D2T	BWR-Bag	Background IoU (%)	Crop row IoU (%)	Furrow IoU (%)	Obstacle IoU (%)	mIoU (%)	mDice (%)
Baseline (PIDNet-S)				91.88	92.44	91.43	79.60	88.84	94.00
+CA-MKIR	✓			93.05	93.35	93.00	87.42	91.71	95.65
+SaE-D2T		✓		93.00	93.95	93.40	86.30	91.66	95.62
+BWR-Bag			✓	93.20	93.55	93.35	87.05	91.79	95.70
+CA-MKIR + SaE-D2T	✓	✓		**93.62**	93.81	**93.47**	87.90	92.20	95.92
+CA-MKIR + BWR-Bag	✓		✓	93.17	93.62	93.18	88.05	92.00	95.82
+SaE-D2T + BWR-Bag		✓	✓	93.18	93.58	93.10	87.54	91.85	95.73
**Agri-PIDNet-S (Full)**	✓	✓	✓	93.31	**94.06**	93.45	**88.67**	**92.37**	**96.02**

*Note:* A check mark indicates that the corresponding module was included. All configurations were evaluated as seed-0 single-run point estimates under the same protocol. Bold values indicate the highest result in each metric column.

Regarding the overall metrics, the original PIDNet-S achieved an mIoU of 88.84%. The IoU values for the background, crop-row, and furrow classes all exceeded 91%, whereas the obstacle IoU was only 79.60%. These results indicate that the dominant region classes already had a strong segmentation baseline in seedling-stage cotton-field scenes, leaving relatively limited room for further improvement in the overall mIoU. In contrast, small-scale obstacles and regions with complex boundaries remained the principal challenges limiting model performance.

After introducing CA-MKIR, the mIoU increased to 91.71%, representing an improvement of 2.87 percentage points over the original PIDNet-S. The obstacle IoU increased from 79.60% to 87.42%, an improvement of 7.82 percentage points, which was the largest obstacle-class gain among the three single-module variants. Under this single-run ablation, the observed pattern is consistent with the intended role of CA-MKIR in representing fine-grained shallow features, including cotton-plant edges, furrow textures, and small-scale obstacle contours.

After introducing SaE-D2T, the model achieved an mIoU of 91.66% and an mDice of 95.62%. The crop-row IoU increased from 92.44% to 93.95%, while the furrow IoU increased from 91.43% to 93.40%, corresponding to improvements of 1.51 and 1.97 percentage points, respectively. Compared with the other single-module variants, SaE-D2T produced larger point-estimate gains for crop-row and furrow regions, which exhibit long-range continuous structures. This pattern is consistent with the module’s intended role in modeling distant row-oriented structures, perspective-convergence relationships, and overall furrow layouts.

When BWR-Bag was introduced alone, the model achieved a background IoU of 93.20% and an mIoU of 91.79%, the highest point estimates for these two metrics among the three single-module variants. Its furrow and obstacle IoUs were 93.35% and 87.05%, corresponding to increases of 1.92 and 7.45 percentage points over the baseline, respectively, although they were not the highest single-module values for these classes.

All three two-module configurations exceeded the baseline, with mIoU values ranging from 91.85% to 92.20% and mDice values ranging from 95.73% to 95.92%. CA-MKIR + SaE-D2T achieved the highest two-module mIoU (92.20%) and also produced the highest background and furrow IoUs among all eight configurations (93.62% and 93.47%, respectively). CA-MKIR + BWR-Bag achieved the highest two-module obstacle IoU of 88.05%. These results show that the joint effects were metric dependent rather than uniformly additive across classes.

The complete Agri-PIDNet-S achieved crop-row and obstacle IoUs of 94.06% and 88.67%, respectively, together with the highest aggregate scores of 92.37% mIoU and 96.02% mDice. Relative to the best single-module configuration, the complete model increased mIoU and mDice by 0.58 and 0.32 percentage points, respectively; relative to the best two-module configuration, the corresponding increases were 0.17 and 0.10 percentage points. The complete model did not produce the highest point estimate for every class, because CA-MKIR + SaE-D2T yielded slightly higher background and furrow IoUs. The full-factorial results therefore support complementary performance patterns across the three modules and show that their joint use provides the best overall point-estimate trade-off under the evaluated protocol. Because each ablation configuration was evaluated in a single run, these differences are interpreted descriptively rather than as statistically significant interaction effects.

### Ablation study of internal components

To further evaluate the necessity of the key internal components within each proposed module, component-level ablation experiments were conducted for CA-MKIR, SaE-D2T, and BWR-Bag. Specifically, coordinate attention was removed from CA-MKIR to obtain MKIR w/o CA; the multi-branch channel recalibration (MBCR) module was removed from SaE-D2T to obtain D2T w/o MBCR; and the window-attention refinement unit was removed from BWR-Bag to obtain BWR-Bag w/o Window Attn. Except for the component being removed, all other network structures and training settings were kept unchanged. The results are presented in Table 5.

**Table 5:** Internal-component ablation results.

Variant	Background IoU (%)	Crop row IoU (%)	Furrow IoU (%)	Obstacle IoU (%)	mIoU (%)	mDice (%)
MKIR w/o CA	91.91	92.81	92.00	85.25	90.49	94.98
**CA-MKIR**	**93.05**	**93.35**	**93.00**	**87.42**	**91.71**	**95.65**
D2T w/o MBCR	90.90	92.95	91.59	84.89	90.08	94.75
**SaE-D2T**	**93.00**	**93.95**	**93.40**	**86.30**	**91.66**	**95.62**
BWR-Bag w/o Window Attn	91.18	92.73	91.97	85.60	90.37	94.92
**BWR-Bag**	**93.20**	**93.55**	**93.35**	**87.05**	**91.79**	**95.70**

*Note:* Bold values indicate the complete version of each module.

Because the background, crop-row, and furrow classes already achieved high IoU values in the baseline model, removing a single internal component produced only a relatively moderate change in the overall mIoU. Therefore, this section focuses primarily on the targeted effects of each component on specific semantic classes and spatial regions, particularly obstacles, crop-row–furrow structures, and areas prone to background misclassification.

The internal ablation results for CA-MKIR show that removing coordinate attention resulted in an mIoU of 90.49% and an obstacle IoU of 85.25%. After coordinate attention was restored, the mIoU increased to 91.71% and the obstacle IoU increased to 87.42%, corresponding to improvements of 1.22 and 2.17 percentage points, respectively. These single-run differences are consistent with the intended use of coordinate attention for directional and positional modeling of cotton-plant edges and small-scale obstacle contours.

For SaE-D2T, removing MBCR resulted in an mIoU of 90.08% and an mDice of 94.75%. With the complete SaE-D2T module, these metrics increased to 91.66% and 95.62%, respectively. The crop-row IoU increased from 92.95% to 93.95%, while the furrow IoU increased from 91.59% to 93.40%. These single-run differences are consistent with the intended role of MBCR in channel-wise selection of deep semantic features and preservation of long-range crop-row and furrow structures.

For BWR-Bag, removing the window-attention refinement unit resulted in an mIoU of 90.37% and an mDice of 94.92%. The complete BWR-Bag increased the background IoU from 91.18% to 93.20%, an improvement of 2.02 percentage points, while increasing the obstacle IoU from 85.60% to 87.05%. The larger background-class difference is consistent with the intended refinement of locally ambiguous regions along furrow boundaries, around field facilities, and within non-operational areas.

Overall, restoring each internal component produced higher point estimates than the corresponding component-removed variant, although the largest changes occurred in different classes. Coordinate attention was associated with higher obstacle and overall IoU, MBCR with higher crop-row and furrow IoU, and window-attention refinement with higher background and obstacle IoU. These single-run ablations support distinct functional roles for the internal components but are not interpreted as estimates of deterministic effects.

### Ablation study of the loss functions

With the network architecture fixed, the contribution of each term in the composite loss was further investigated. The complete Agri-PIDNet-S with the full composite loss was used as the reference, and a one-at-a-time ablation strategy was used, in which the Dice loss, Lovász loss, and auxiliary boundary loss were individually removed. The cross-entropy loss was retained in all variants as the basic pixel-level semantic supervision. All other training settings, dataset splits, and evaluation procedures remained unchanged. The results are presented in Table 6.

**Table 6:** Results of the loss-function ablation experiments.

Variant	Background IoU (%)	Crop row IoU (%)	Furrow IoU (%)	Obstacle IoU (%)	mIoU (%)	mDice (%)
w/o Dice	91.88	93.06	92.21	85.22	90.59	95.03
w/o Lovász	92.28	92.85	92.28	83.68	90.27	94.84
w/o Boundary	91.39	92.73	91.78	84.81	90.18	94.81
**Full loss**	**93.31**	**94.06**	**93.45**	**88.67**	**92.37**	**96.02**

*Note:* Bold values indicate the best result in each metric column.

Overall, the full composite loss yielded the highest point estimates among the evaluated loss configurations, with an mIoU of 92.37% and an mDice of 96.02%. Removing any individual loss term produced lower scores in the corresponding single-run ablation. Because the background, crop-row, and furrow classes already achieved relatively high IoU values, removing a single loss term produced only moderate changes in the overall mIoU. The differences among the loss configurations were more evident for critical targets and regions, particularly obstacles and class boundaries.

After removing the Dice loss, the mIoU decreased to 90.59%, the mDice decreased to 95.03%, and the obstacle IoU declined from 88.67% to 85.22%. This pattern is consistent with the role of Dice loss in promoting overlap agreement between predicted and ground-truth regions. Its region-overlap objective partially overlaps with that of the Lovász loss, which may explain why this variant exhibited the smallest overall decrease among the three loss ablations.

After removing the Lovász loss, the obstacle IoU decreased to 83.68%, representing the largest reduction in obstacle performance among the three ablation variants, while the mIoU decreased to 90.27%. This pattern is consistent with the IoU-oriented objective of the Lovász loss and its intended contribution to the underrepresented obstacle class.

After removing the auxiliary boundary loss, the mIoU decreased to 90.18%, the mDice decreased to 94.81%, and the obstacle IoU decreased to 84.81%. Lower point estimates were also observed for the background and furrow classes. This pattern is consistent with the intended role of auxiliary boundary supervision in guiding the D branch toward crop-row edges, furrow boundaries, and obstacle contours.

The four loss terms target pixel-level classification, regional overlap consistency, IoU-oriented optimization, and boundary-structure constraints, respectively. The cross-entropy loss provides basic semantic supervision, the Dice loss promotes regional overlap consistency, the Lovász loss aligns optimization more closely with mIoU, and the auxiliary boundary loss constrains the boundary representation of the D branch. Under the evaluated settings, their joint use yielded the highest observed overall and obstacle-class scores among the tested loss configurations.

### Analysis of obstacle scale sensitivity and misclassification patterns

To further characterize Agri-PIDNet-S performance in seedling-stage cotton fields, the scale sensitivity of the safety-critical obstacle class and the overall pattern of model misclassifications were analyzed in addition to overall accuracy. First, obstacle instances were extracted as connected components from the ground-truth masks of the test set. To reduce interference from extremely small isolated components in the instance-scale statistics, connected components with areas smaller than 256 pixels were excluded only from the obstacle-scale analysis; this filtering was not applied when calculating the confusion matrix or the pixel-level evaluation metrics reported above. The remaining obstacle instances were then divided into small, medium, and large groups according to the 33rd and 67th percentiles of their areas. The detection rate was defined as the proportion of obstacle instances for which more than 50% of the ground-truth pixels were predicted as obstacles. Pixel recall represented the proportion of pixels within obstacle instances of each size group that were correctly classified as obstacles. Finally, a row-normalized confusion matrix was calculated using all pixels in the test set to identify the principal sources of confusion among the four semantic classes. The resulting scale-wise metrics and confusion matrix are presented in Fig 10.

**Fig 10:**
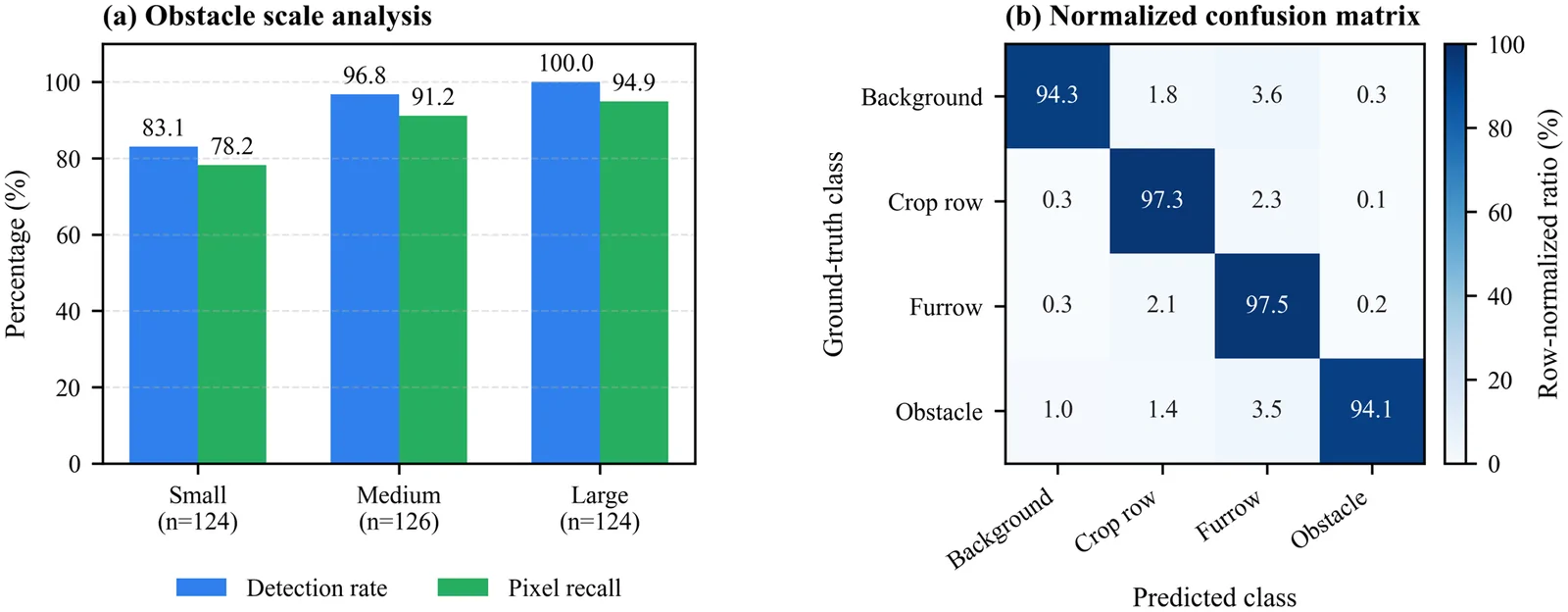
Obstacle-scale sensitivity and pixel-level error structure. (a) Detection rate and pixel recall for small, medium, and large obstacle instances; and (b) row-normalized confusion matrix on the cotton-field test set.

As shown in Fig 10(a), Agri-PIDNet-S achieved its highest scale-specific results for large obstacles, with a detection rate of 100.0% and a pixel recall of 94.9%. For medium-sized obstacles, the detection rate and pixel recall reached 96.8% and 91.2%, respectively. For small obstacles, these metrics decreased to 83.1% and 78.2%, indicating that distant or small-area obstacles remain challenging in cotton-field semantic segmentation. Overall, the observed obstacle-perception scores increased with object size, consistent with the greater susceptibility of small objects to limited spatial resolution, boundary ambiguity, and background interference.

Fig 10(b) presents the row-normalized confusion matrix for the test set. All four classes exhibited high diagonal values, with correct classification rates of 94.3%, 97.3%, 97.5%, and 94.1% for the background, crop-row, furrow, and obstacle classes, respectively. The obstacle-class diagonal value of 94.1% agrees with the obstacle recall of 94.09% reported in Table 2. The off-diagonal entries show that the main confusion occurred between the background and furrow, crop-row and furrow, and obstacle and furrow classes. Specifically, 3.6% of background pixels, 2.3% of crop-row pixels, and 3.5% of obstacle pixels were misclassified as furrows. These errors were mainly associated with bare-soil regions, damaged plastic mulch, vegetation edges, and transition areas near obstacle boundaries, reflecting the visual similarity between adjacent ground-surface classes and the irregular boundaries characteristic of seedling-stage cotton fields.

Further analysis showed that 65.08% of all misclassified pixels were located within a three-pixel neighborhood of the ground-truth class boundaries. This finding indicates that the residual errors of Agri-PIDNet-S were concentrated primarily in boundary-transition regions rather than arising from systematic misclassification across large semantic areas. The result is consistent with the design objectives of the proposed network: SaE-D2T enhances the understanding of global crop-row and furrow structures in deep features, whereas BWR-Bag uses boundary information from the D branch to guide the fusion of the P and I branches and further refines local boundaries through window attention.

Overall, the obstacle results were scale dependent, and the remaining errors were concentrated mainly in complex boundary regions. These findings identify small obstacles and boundary transitions as the principal targets for further improvement.

### Zero-shot cross-crop external evaluation

To assess structural transfer outside the cotton source domain, a small-scale cross-crop external test set was constructed from 69 independently captured still images: 24 tomato, 23 watermelon, and 22 maize images. The images were collected by the authors from May to early June 2026 at the Sanping Farm teaching and practical-training base of Xinjiang Agricultural University using an iPhone 15 Pro at a native resolution of 5712×4284 pixels. They were not extracted from continuous or burst sequences, and repeated adjacent frames or highly correlated viewpoints were not included. At this collection site, access and non-invasive image acquisition were covered by the university’s routine teaching and research management arrangements; therefore, no separate field-access permit or permit number was required. The author-produced images and annotations were available for research use and public release.

All 69 images had pixel-wise ground-truth masks created in LabelMe using the same four-class label system as the cotton dataset: unlabeled pixels were assigned to background, and the explicit polygon labels were crop_row, furrow, and obstacle. The annotations followed the same quality-control workflow: two annotators with computer-vision and agricultural-engineering backgrounds performed self-checking and reciprocal cross-checking, followed by final semantic review by an agricultural engineering specialist. Obstacle pixels occurred in 18 of 24 tomato images, all 23 watermelon images, and 5 of 22 maize images. Manual instance counting identified 83, 109, and 13 physical obstacle objects, respectively, for a total of 205.

The cross-crop images were used only for external testing and were not involved in training, parameter tuning, model selection, or fine-tuning. The fixed seed-0 Agri-PIDNet-S model used for the cotton comparison was directly applied to all three crops. Input preprocessing and prediction restoration followed the cotton-field test protocol described above. The class-wise IoU results and the structural and obstacle metrics are reported in Tables 7 and 8, respectively.

**Table 7:** Zero-shot cross-crop IoU results with image-level 95% confidence intervals.

Crop	*N*	Background IoU (%)	Crop row IoU (%)	Furrow IoU (%)	Obstacle IoU (%)	mIoU (%)
Tomato	24	94.06 (89.69–96.39)	92.96 (91.46–94.27)	93.63 (93.03–94.25)	71.03 (68.49–72.70)	87.92 (86.46–88.85)
Watermelon	23	77.86 (67.87–85.32)	71.00 (68.65–73.59)	90.71 (89.82–91.52)	78.35 (72.57–81.75)	79.48 (77.32–81.08)
Maize	22	82.56 (76.59–86.59)	83.55 (79.53–87.00)	84.73 (82.03–87.49)	45.32 (25.23–59.34)	74.04 (69.32–77.63)
Overall	69	84.93 (79.86–88.84)	84.19 (81.56–86.53)	89.76 (88.54–90.92)	68.16 (60.52–75.77)	81.76 (79.54–83.97)

*Note:* Values are point estimates with image-level 95% CIs in parentheses. Overall point estimates pool the pixels from all 69 images; Overall CIs use unstratified image resampling from the pooled set.

**Table 8:** Structural and obstacle results with image-level 95% confidence intervals.

Crop	*N*	Structural mIoU (%)	Obstacle precision (%)	Obstacle recall (%)	Obstacle F1-score (%)
Tomato	24	93.55 (91.63–94.83)	96.81 (95.80–97.27)	72.74 (70.31–74.57)	83.06 (81.30–84.19)
Watermelon	23	79.86 (76.06–83.00)	82.53 (77.41–85.53)	93.93 (91.85–95.09)	87.86 (84.11–89.96)
Maize	22	83.61 (80.91–85.78)	79.06 (32.01–91.61)	51.50 (38.69–78.02)	62.37 (40.30–74.48)
Overall	69	86.29 (83.98–88.27)	85.03 (79.39–88.75)	77.46 (67.96–87.98)	81.07 (75.40–86.21)

*Note:* Structural mIoU is the mean IoU of the background, crop-row, and furrow classes. Values are point estimates with image-level 95% CIs in parentheses. Only 5 maize images contained obstacles, comprising 13 physical instances; the maize obstacle metrics are therefore reported as reference results.

Using the source-domain cotton test-set structural mIoU of 93.61% as a reference, Tables 7 and 8 show that, without cross-crop fine-tuning, structural mIoU was 93.55% (95% CI: 91.63–94.83%) for tomato, 79.86% (76.06–83.00%) for watermelon, and 83.61% (80.91–85.78%) for maize. The watermelon furrow IoU remained high at 90.71%, whereas its crop-row IoU was 71.00%, indicating sensitivity to differences in plant size, coverage pattern, and ground-surface appearance.

Across all 69 external images, structural mIoU was 86.29% (83.98–88.27%) and four-class mIoU was 81.76% (79.54–83.97%). Obstacle performance varied more strongly across crops: obstacle IoU was 71.03% for tomato, 78.35% for watermelon, and 45.32% for maize. The maize obstacle estimates had wide intervals, including an obstacle IoU CI of 25.23–59.34% and an F1-score CI of 40.30–74.48%, consistent with the fact that only 5 maize images contained 13 obstacle instances. Therefore, the maize obstacle results are treated as reference values rather than evidence of stable cross-crop obstacle performance. Overall, the results provide preliminary evidence of zero-shot transfer for crop-row and furrow structure, while obstacle transfer remains uncertain. Representative predictions for the three crop types are shown in Fig 11.

**Fig 11:**
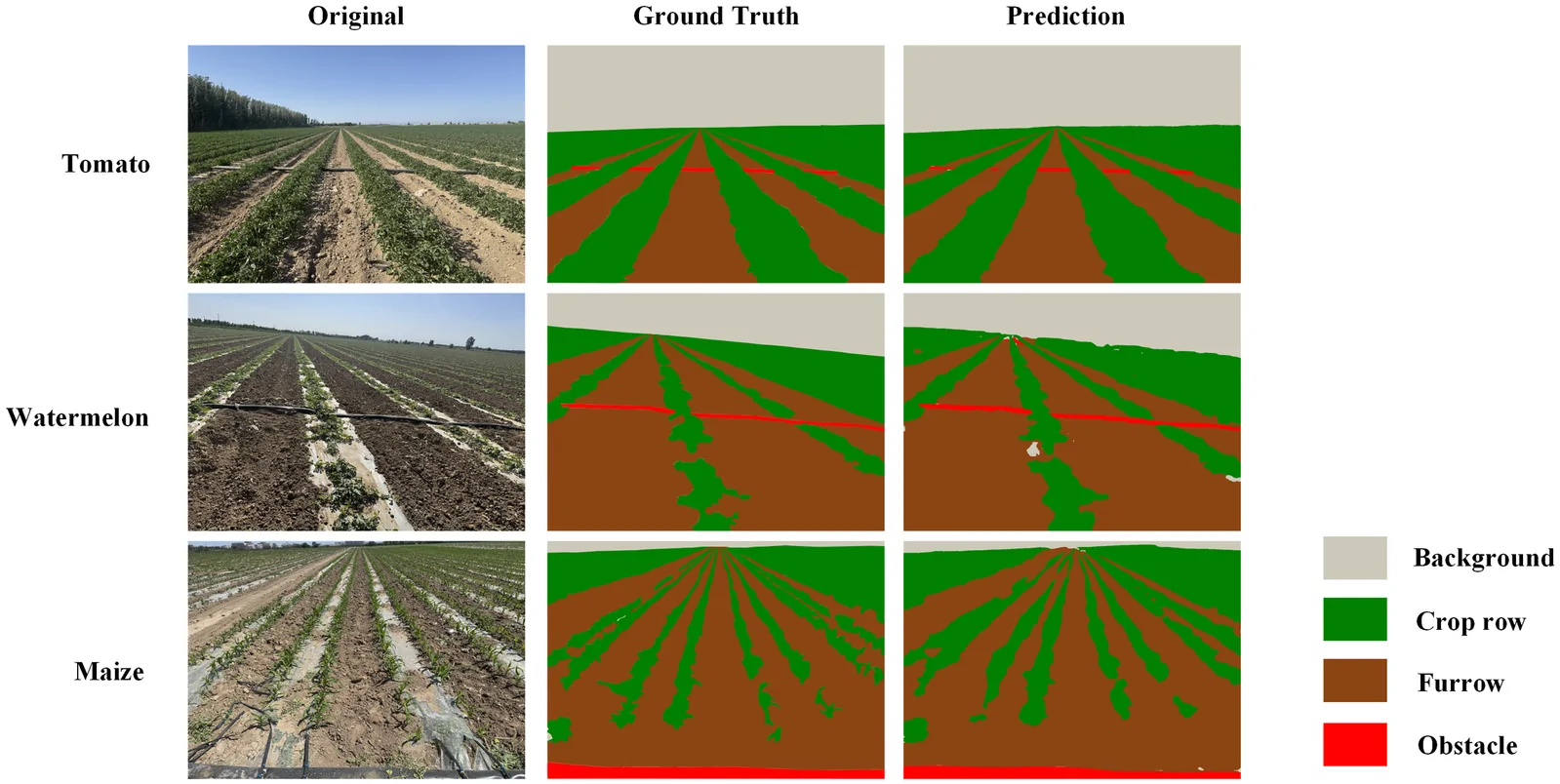
Zero-shot segmentation results of Agri-PIDNet-S in different row-crop scenes. From top to bottom, the rows correspond to tomato, watermelon, and maize scenes; from left to right, the columns show the original images, ground-truth masks, and prediction results.

Fig 11 further illustrates the zero-shot predictions across the three crop scenes. In the tomato example, the predicted crop-row and furrow regions closely followed the ground-truth structures. In the watermelon example, the principal furrow structures and most of the transverse irrigation pipe were segmented despite interference from bare soil and plastic mulch. In the maize example, the prediction broadly distinguished the row-oriented crop and furrow regions, although misclassifications remained along local boundaries and in narrow distant areas.

Taken together, the quantitative and visual results provide preliminary evidence that Agri-PIDNet-S can retain some crop-row and furrow structure information outside the cotton source domain. However, this experiment represents only a small-scale external evaluation, with limited sample size, crop diversity, and obstacle distribution, and does not establish broad cross-crop generalization. Future work should expand the dataset to include additional regions, growth stages, and crop types and investigate few-shot fine-tuning or domain-adaptation methods to improve performance in complex cross-crop scenarios.

## Discussion

This study addressed the need for unified pixel-level perception of crop rows, furrows, and obstacles during autonomous inter-row operations in seedling-stage cotton fields. The dataset statistics show that obstacles accounted for only 2.60% of all pixels but appeared in 95.77% of the images, indicating that they were frequent yet spatially small targets. Although the baseline PIDNet-S achieved IoU values above 91% for the background, crop-row, and furrow classes, its obstacle IoU was only 79.60%. In the model-comparison experiment, Agri-PIDNet-S increased the overall mIoU to 92.37% and the obstacle IoU to 88.67%, while retaining 8.048 million parameters and 19.161 GFLOPs. Across three training seeds, mIoU was 92.35 ± 0.03% and obstacle IoU was 88.54 ± 0.14%, indicating limited run-to-run variation under the fixed protocol. The observed gains were therefore obtained through task-oriented architectural changes rather than an increase in model size.

The full-factorial module ablation produced metric-dependent patterns across the eight configurations. CA-MKIR provided the largest single-module increase in obstacle IoU, SaE-D2T produced the highest single-module crop-row and furrow IoUs, and BWR-Bag produced the highest single-module mIoU. All two-module configurations exceeded the baseline, while the complete model achieved the highest overall mIoU and mDice and the highest crop-row and obstacle IoUs. CA-MKIR + SaE-D2T retained slightly higher background and furrow IoUs than the complete model. These single-run results support complementary performance patterns across the modules without establishing statistically significant interaction effects. In the loss ablation, removing the Lovász term produced the largest observed decrease in obstacle IoU.

The obstacle-scale and error analyses provide additional insight into the remaining prediction behavior. Agri-PIDNet-S achieved high detection rates for medium- and large-scale obstacles, whereas small obstacles remained more sensitive to spatial resolution and background interference. Moreover, 65.08% of all misclassified pixels occurred within three pixels of the ground-truth boundaries, showing that most residual errors were concentrated in transition regions rather than large semantic areas. The obstacle precision and recall of 93.89% and 94.09%, respectively, represent a balanced result in the seed-0 evaluation. Together with an inference speed of 155.67 FPS under the reported RTX 4090 configuration, these results provide computational-efficiency evidence for real-time perception under the reported hardware and evaluation conditions.

Without fine-tuning, the model achieved an overall structural mIoU of 86.29% (95% CI: 83.98–88.27%) across the tomato, watermelon, and maize images. The differences among crops reflect variation in plant morphology, canopy coverage, and ground appearance and provide only preliminary evidence that some crop-row and furrow structure information transfers beyond the cotton source domain. The wide maize obstacle intervals further show that obstacle transfer cannot be established from the present external sample. The internal evaluation remains limited to one farm, one region, one seedling-stage acquisition period, the lighting conditions encountered during that period, and the three handheld devices listed in the “Image acquisition and screening” subsection; it should not be interpreted as evidence of generalization beyond these conditions. The external dataset is also limited in sample size, crop coverage, and obstacle distribution. Future work will therefore extend validation across additional farms, regions, seasons, growth stages, lighting conditions, acquisition platforms, and crop types and evaluate the model on edge-computing and mobile agricultural platforms.

## Conclusions

This study constructed a four-class pixel-wise annotated dataset for seedling-stage cotton fields and developed Agri-PIDNet-S for the unified perception of crop rows, furrows, and obstacles. CA-MKIR, SaE-D2T, and BWR-Bag were introduced to enhance shallow fine-grained features, deep global row structures, and boundary-guided feature fusion, respectively. On the cotton-field test set, Agri-PIDNet-S achieved an mIoU of 92.37% and an mDice of 96.02% in the model-comparison experiment, while three training seeds yielded an mIoU of 92.35 ± 0.03%. Compared with PIDNet-S under the same comparison protocol, the model improved mIoU by 3.53 percentage points and increased obstacle IoU from 79.60% to 88.67%. It required 8.048 million parameters and 19.161 GFLOPs and reached 155.67 FPS under the reported hardware configuration. Zero-shot external evaluation yielded a structural mIoU of 86.29% across tomato, watermelon, and maize fields, providing preliminary evidence of cross-crop transferability. Overall, Agri-PIDNet-S provides an accurate and computationally efficient semantic-perception basis for cotton-field inter-row operations.
